# Photochromism of Dihydroazulene Embedded in Solid Polymer Matrices

**DOI:** 10.1021/acs.jpcb.6c04116

**Published:** 2026-09-10

**Authors:** Roman Golovchak, Liudmila Loghina, Joshua Rye, Emmabeth Parrish Vaughn, Andriy Kovalskiy, Keira Scott, Calleway Schmidt, Roman Jambor, Miroslav Vlcek

**Affiliations:** † Department of Physics, Engineering and Astronomy, 2536 Austin Peay State University, Clarksville, Tennessee 37044, United States; ‡ Center of Materials and Nanotechnologies, Faculty of Chemical Technology, 48252 University of Pardubice, Pardubice 530 02, Czech Republic; § Department of General and Inorganic Chemistry, Faculty of Chemical Technology, University of Pardubice, Pardubice 532 10, Czech Republic

## Abstract

Photochromic dihydroazulene/vinylheptafulvene systems have been extensively investigated in solution, whereas their behavior in solid polymer matrices remains much less understood despite their relevance for photonic and optically responsive devices. Here, a phenyl-substituted dihydroazulene derivative (Ph-DHA) was embedded in nine optically transparent polymer matrices that differ in polarity, chemical functionality, rigidity, and segmental mobility. The resulting films were examined by in situ optical transmission measurements under continuous 365 nm irradiation and during subsequent thermal recovery. UV exposure induced pronounced coloration of all films, accompanied by a red shift of the optical absorption edge from approximately 2.7–2.9 eV to 1.8–1.9 eV, indicating formation of the photoinduced vinylheptafulvene form in the solid state. The initial photoinduced changes in absorbance followed a stretched exponential time dependence with the stretch exponent *β* approaching unity (pure exponential). The minimal time constants (fastest responses) were observed in the 460–500 nm spectral region. Thermal recovery at elevated temperatures was also adequately described by a stretched exponential function but with greater *β* variation (0.6–1.0). The characteristic relaxation times showed weaker spectral dependence and generally decreased upon heating. No simple correlation between the kinetic parameters and any single polymer property, such as polarity, molecular weight, or chemical functionality, was observed. Instead, the results suggest that a broad distribution of local microenvironments and relaxation pathways within the polymer host governs the rate of photochromic response of Ph-DHA in solid films. This systematic comparison demonstrates that Ph-DHA retains robust and thermally reversible photochromism across chemically diverse solid polymer matrices and provides a phenomenological basis for designing polymer-based photoresponsive films.

## Graphical abstract

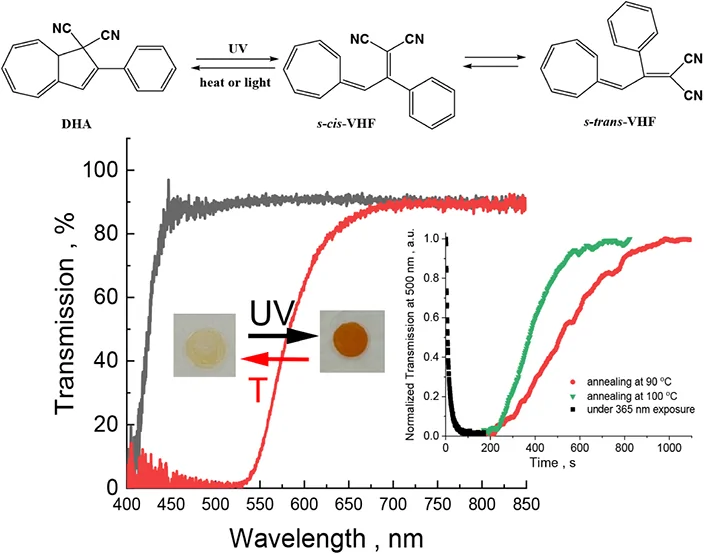

## 1 Introduction

Artificial chromic systems, where the change in color is caused by light exposure, are considered key components in many applications, including photoswitches, dynamic photopatterning, stimuli sensors, and even organic transistors.^1–5^ The most common reported photochromic materials are designed and synthesized based on several types of molecular structures, including diarylethene, spiropyran, dihydroazulene, and similar compounds.^3,4^ The dihydroazulene (DHA) photochromes possess high sensitivities and temporal-spatial resolution, which makes them attractive candidates for solar energy harvesting^6^ and information storage systems.^7^ Recently, photochromic materials based on DHA have attracted substantial interest due to the significant spectral difference in absorption maxima between the two isomers interconverted by light, heat, or Lewis acids.^8–10^ It is well known that upon light absorption, the DHA molecule is excited from the electronic ground state (S0) to the lowest-lying singlet excited state (S1), corresponding to a ππ* transition.^11–13^ The excited molecule undergoes a photoinduced high quantum yield ring-opening reaction, which leads to the formation of the metastable s-cis-vinylheptafulvene (VHF) isomer through an S1/S0 conical intersection. The cis-VHF isomer can either undergo a reverse thermal ring-closure reaction with a relatively high energy barrier (18–24 kcal/mol) to restore the DHA or transform into the metastable s-trans-VHF isomer with an energy barrier of 6 kcal/mol via a thermal cis–trans isomerization process.^11–13^ The experimental studies indicate that the ring-opening reaction is strongly dependent on the chemical environment and substitution of the rings.^12^ The speed of the switch between (partially) opaque and transparent states can be in the order of nanoseconds.^13–15^

Recent research on DHA/VHF photoswitches has moved beyond the fundamental characterization of the photoinduced ring-opening and thermal ring-closing reactions toward deliberate control of switching kinetics and integration of the photochromic couple into functional molecular and materials systems. Structural modification of the DHA core has been used to tune the absorption properties and, particularly, the lifetime of the metastable VHF state; for example, incorporation of an ethynyl substituent at the C2 position was shown to prolong the VHF half-life from several hours to several weeks at room temperature.^16^ DHA units have also been incorporated into photofluorochromic aggregation-induced-emission systems for rewritable multidimensional information encoding,^17^ coupled to fluorophores for light-controlled emission, and employed as photo-/thermochemical elements in light-gated organic transistors.^5^ More recently, DHA/VHF switching has been demonstrated in aqueous peptide conjugates^18^ and in porous metal-organic-framework hosts,^19^ illustrating that the switching behavior can be retained in chemically and structurally very different environments. In particular, the pronounced dependence of solid-state DHA/VHF switching on the nature and confinement characteristics of the host highlights the increasing importance of the surrounding matrix as an active parameter in controlling the photochromic response.

The interconversions can occur when the photochrome is in both solid and dissolved forms. Solvation of the photochromic molecule by both solvent molecules and the polymer chain has a significant effect on the kinetics of interphase transformation, as well as the stabilization of each form.^20^ Nevertheless, the detailed studies of DHA photochromism have only been performed for solutions, while the photochromism of DHA embedded into solid-state polymer matrices (desired for device applications) has not been studied comprehensively. In contrast to a solution, where molecular motion and thermal equilibration dominate the photochromic response, the solid-state polymer environment imposes strong spatial and dynamic constraints on the photoactive molecule. Such confinement is expected to affect not only the stability of the photoinduced VHF form, but also the apparent kinetics of its formation and relaxation, as shown in the example of DHA embedded into poly­(methyl methacrylate) (PMMA) and poly­(bisphenol A carbonate) (PC) solid-state matrices.^21^

When a photochromic molecule is dispersed in a solid polymer film, the host matrix acts not only as a mechanically stable support but also as a local microenvironment for the embedded chromophore. Even in the absence of covalent bonding, polymer polarity, free volume, segmental mobility, and weak specific interactions can influence the formation and relaxation of the photoinduced isomer. As a result, the same photochrome may display different coloration efficiency, spectral evolution, and recovery kinetics depending on the matrix in which it is embedded. Understanding this matrix-dependent modulation is therefore essential for translating solution-phase DHA/VHF photochromism into functional solid-state photonic materials.

In this work, a phenyl-substituted dihydroazulene derivative, 2-phenylazulene-1,1­(8a*H*)-dicarbonitrile (Ph-DHA), was embedded into a series of optically transparent polymer matrices differing in chemical structure, polarity, hydrogen-bonding capability, aromatic content, and segmental mobility. The selected hosts include nonpolar hydrocarbon-based matrices, ester-containing polymers, nitrogen-containing polymers, and carbazole-based materials. This set was chosen to provide a broad range of solid-state microenvironments while preserving optical transparency in the visible region. The resulting Ph-DHA/polymer films were examined by in situ transmission spectroscopy under continuous 365 nm UV irradiation. Millisecond-resolved photoinduced optical changes were recorded at multiple wavelengths spanning the fundamental optical absorption edge, allowing the spectral dispersion of the coloration kinetics to be followed for each polymer/Ph-DHA composite. In addition, thermal recovery kinetics were measured at 90 and 100 °C to evaluate the delayed relaxation of the photoinduced state in the solid polymer environment. The study shows that Ph-DHA retains pronounced and thermally reversible photochromism in all investigated solid matrices. UV exposure induces a substantial red shift of the apparent absorption edge consistent with formation of the VHF state, and the time-dependent optical changes can be still approximated by simple first-order kinetics. Conversely, the thermal recovery absorbance responses are described by stretched exponential behavior. The comparison across polymer hosts reveals no simple correlation with a single polymer property, suggesting that the solid-state photochromic response is governed by a broad distribution of local microenvironments and relaxation processes.

## 2 Experimental Section

### 2.1 Materials

All reagents and solvents for Ph-DHA synthesis were obtained from commercial suppliers and used as received unless otherwise noted. Ammonium acetate, Na_2_SO_4_ and solvents were acquired from Penta (Czech Republic). Acetophenone and nitrosonium tetrafluoroborate were purchased from BLDpharm (Germany). Malononitrile and tropylium tetrafluoroborate were obtained from Thermo Fisher (Czech Republic). Reactions were monitored by thin-layer chromatography (TLC) on silica gel 60 F_254_ plates. Column chromatography was carried out using silica gel (60 Å, 70–230 mesh). All products were dried under reduced pressure and stored in a dark environment.

#### 2.1.1 Synthesis of 2-(1-Phenylethylidene)­malononitrile 1

A solution of acetophenone (8.10 g, 67.4 mmol) in toluene (120 ml) was treated with malononitrile (11.16 g, 169 mmol) and saturated ammonium acetate solution (50 ml). The reaction mixture was refluxed using a Dean-Stark apparatus until the separation of water ceased (≈15 h). The conversion of the starting material was monitored by TLC (petroleum ether/ethyl acetate = 9:1). After completion, the mixture was cooled to room temperature, diluted with diethyl ether, and washed twice with water. The organic layer was dried over anhydrous Na_2_SO_4_ and concentrated under reduced pressure. The crude product was purified by column chromatography on silica gel (petroleum ether/ethyl acetate = 9:1) to give 2-(1-phenylethylidene)­malononitrile **1** as white crystalline solid (9.1 g, 79%). ^1^H NMR (500.13 MHz, CDCl_3_), δ: 7.49–7.45 (m, 5H), 2.56 (s, 3H). ^13^C NMR (125.76 MHz, CDCl_3_), δ: 175.68; 135.83; 132.25; 129.07; 127.33; 112.85; 112.76; 84.52; 24.25.

#### 2.1.2 Synthesis of 2-Phenylazulene-1,1­(8aH)-Dicarbonitrile 3

Under an argon atmosphere, tropylium tetrafluoroborate (2.00 g, 11.2 mmol) was added to a solution of compound **1** (1.80 g, 10.7 mmol) in dry dichloromethane (80 mL). The reaction mixture was cooled to −78 °C, and a solution of triethylamine (1.53 mL, 11.0 mmol) was added dropwise over 1 h. The mixture was stirred at −78 °C for an additional 30 min, then the cooling bath was removed, and the reaction was allowed to warm to 0 °C. The reaction was quenched with 1% HCl, and the layers were separated. The organic phase was extracted with dichloromethane, and the combined organic extracts were washed with water and brine, dried over anhydrous Na_2_SO_4_, and concentrated under reduced pressure. The crude semisolid **2** was used in the next step without further purification.

The crude oil **2** (corresponding to 0.01 mol) was dissolved in dry acetonitrile (60 mL) in a three-neck flask, protected from light and under argon, and the solution was cooled to −10 °C. Nitrosonium tetrafluoroborate (NOBF_4_, 2.34 g, 0.020 mol) was added portion-wise over 30 min. The mixture was stirred at −10 °C for 1 h, then diluted with dry dichloromethane (50 mL) and treated with pyridine (1.13 mL) delivered in 10 mL of dry acetonitrile. The cooling bath was removed, and the reaction was quenched with water. The product was extracted with dichloromethane; the combined organic extracts were washed with water and brine, dried over Na_2_SO_4_, and concentrated in vacuo. The crude residue was purified by flash column chromatography on silica gel (petroleum ether/ethyl acetate = 9:1) to give 2-phenylazulene-1,1­(8aH)-dicarbonitrile **3** as a beige crystalline solid (1.9 g, 74% overall yield for the two stages). ^1^H NMR (500.13 MHz, CDCl_3_), δ: 7.69–7.68 (d, ^3^ *J*_H,H_ = 7.70 Hz, 2H), 7.43–7.37 (m, 3H), 6.83 (s, 1H), 6.52–6.49 (dd, ^3^ *J*_H,H_ = 11.25, 6.45 Hz, 1H), 6.43–6.40 (dd, ^3^ *J*_H,H_ = 11.25, 6.45 Hz, 1H), 6.29–6.23 (m, 2H), 5.78–5.75 (dd, ^3^ *J*_H,H_ = 10.21, 3.22 Hz, 1H), 3.73 (t, ^4^ *J*_H,H_ = 1.47 Hz, 1H). ^13^C NMR (125.76 MHz, CDCl_3_), δ: 140.03; 138.59; 132.32; 130.86; 130.83; 130.37; 129.99; 129.21; 127.60; 126.18; 120.94; 119.39; 115.13; 112.71; 51.04; 45.11.

### 2.2 Preparation of Polymer Films with Embedded Ph-DHA Molecules

The films were wet-deposited on microscopy glass slides from a polymer solution and then dried. First, the polymer solutions were prepared by dissolving 0.4 g of a chosen photo-inert polymer matrix in 2 mL of chloroform (Sigma-Aldrich, USA), stirring, and then leaving them to stand for 24 h. The photo-inert polymers Polystyrene (Mw 35,000), Poly­(ethyl methacrylate) (Mw 377,000), Polystyrene-*block*-polyisoprene-*block*-polystyrene (styrene 17 wt %, Mn 1,900), Poly­(vinyl acetate) (Mw 100,000), Poly­(vinyl butyral-*co*-vinyl alcohol-*co*-vinyl acetate) (Mw 50,000–80,000), Polyvinylpyrrolidone (Mw 60,000), Poly­(4-vinylpyridine) (Mw 60,000), and Poly­(*N*-vinyl carbazole) (Mw 135,600) were obtained from Sigma-Aldrich (Czech Republic). Poly­[9-(2,3-epoxypropyl)­carbazole] (Mw ∼ 2,000) was prepared according to the published method.^22^ The polymer solutions were then mixed in the absence of UV/daylight with 0.02 grams of synthesized Ph-DHA to form a clear photopolymer solution. The as-deposited films were left to dry under a hood for 24 h in the dark at ambient humidity and room temperature. Their thickness was measured by ellipsometry^23^ and was in the range of 1–2 μm. All prepared photopolymer films were sealed in hermetic plastic bags and stored in a dark place at room temperature before and after the measurements.

### 2.3 Characterization

The molecular structure of the prepared Ph-DHA was verified by nuclear magnetic resonance (NMR) spectra, recorded from solutions in CDCl_3_ at 295 K on a Bruker Ascend 500 spectrometer (equipped with a Z-gradient 5 mm Prodigy cryoprobe) at frequencies of 500.13 MHz for ^1^H and 125.76 MHz for ^13^C­{^1^H}, respectively. The solutions were obtained by dissolving approximately 50 mg of Ph-DHA compound in 1.0 mL of CDCl_3_. The values of ^1^H chemical shifts were calibrated to residual signals of CDCl_3_ (δ­(^1^H) = 7.26 ppm). The values of ^13^C chemical shifts were referenced to the signals of CDCl_3_ (δ­(^13^C) = 77.23 ppm). Positive chemical shift values denote shifts to the higher frequencies relative to the standards.

The in situ kinetics of the optical transmission changes were collected (Avasoft 8.4 software) in air with an integration time of 1 ms, simultaneously for a number of fixed wavelengths across the entire fundamental optical absorption edge of the films (420–700 nm), using Avantes AvaSpec-ULS2048XL-2-USB2 (400–1100 nm range) fiber optic spectrometer. The UV irradiation was performed with ThorLabs light emitting diode (LED) at 365 nm wavelength. The irradiation power was 1 mW (power density 1.27 mW/cm^2^), as measured at the sample’s position with Newport 843-R optical power meter equipped with 818-UV/DB sensor. The normalization and fitting of kinetic curves were done in Origin software. A temperature-controlled chamber, Linkam (L-THMS350/EV-4), equipped with quartz windows, was used for in situ optical transmission kinetic measurements at 90 °C and 100 °C temperatures right after UV exposure. The target annealing temperatures were achieved with 30 °C/min constant heating rate, and transmission measurements started immediately after the sample was at the target temperature.

## 3 Results and Discussion

The target compound 2-phenylazulene-1,1­(8aH)-dicarbonitrile (Ph-DHA) was prepared through a three-step synthetic sequence starting from acetophenone, following a modified literature procedure (Figure S1 in Supporting Information Materials).^13,17^ The presence of the gem-dicyano fragment in Ph-DHA is important for its photochromic behavior, as this electron-withdrawing unit contributes to the stabilization of the photoinduced vinylheptafulvene form. Therefore, Ph-DHA represents a suitable model compound for evaluating how a spatially constrained polymer environment affects DHA/VHF photochromism in the solid state.

### 3.1 Molecular Design and Photochromic Transformation of Ph-DHA in Polymer Matrices

The photochromic response of Ph-DHA is based on the reversible interconversion between the closed dihydroazulene (DHA) form and the open vinylheptafulvene (VHF) form (Figure 1).^24^ Upon UV irradiation, the DHA form undergoes a photoinduced ring-opening reaction, producing the more extended π-conjugated VHF isomer. The reverse transformation, VHF → DHA, occurs thermally and is known to be sensitive to both molecular substitution and the surrounding medium. In Ph-DHA, the gem-dicyano fragment contributes to the stabilization of the photoinduced VHF form, making this molecule suitable for investigating delayed recovery and matrix-dependent photochromic behavior in the solid state.^16^

**Figure 1:**
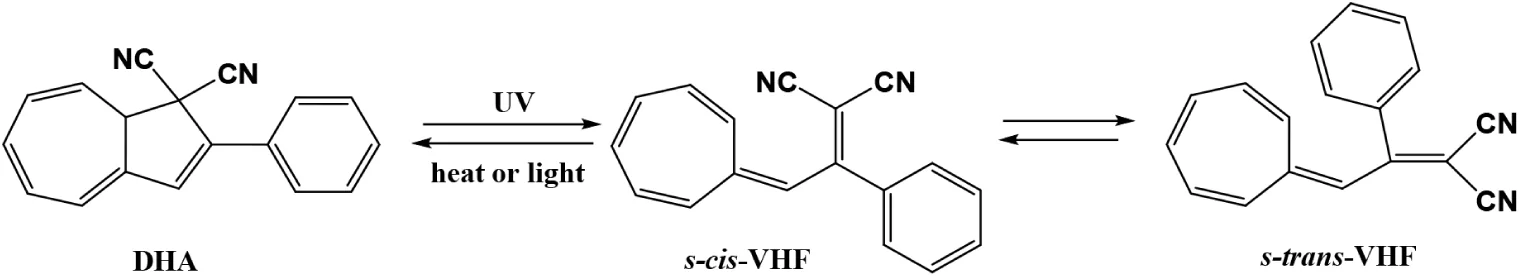
Photoswitching of the dihydroazulene/vinylheptafulvene couple induced by UV light irradiation and temperature exposure.

In the present films, Ph-DHA is physically embedded in the polymer matrices rather than covalently attached to the polymer chains. Therefore, the polymer host is expected to influence the photochromic response mainly through local environmental effects, including polarity, free volume, segmental mobility, rigidity, and weak specific interactions with the photochromic molecule. These factors can affect the efficiency of VHF formation, the stability of the photoinduced state, and the rate of thermal ring closure, while preserving the fundamental DHA/VHF switching mechanism.

The selected polymer matrices provide a broad set of chemically and dynamically different solid-state environments. Nonpolar polystyrene-based matrices, such as PS and PSIS, primarily provide dispersive and aromatic interactions together with mechanical confinement. Ester-containing matrices, including PEMA and PVAc, introduce a more polar environment that may interact with the dicyano fragment through dipolar interactions. PVB contains hydroxyl groups and can provide weak hydrogen-bonding interactions, whereas PVP and P4VP introduce nitrogen-containing polar environments capable of additional dipolar or donor–acceptor interactions. PVK and PEPC represent carbazole-containing hosts, where aromatic interactions and increased rigidity may further influence the local environment of Ph-DHA. PEPC should be regarded as an oligomeric carbazole-containing reference rather than as a directly comparable low-molecular-weight polymer matrix.

This set of hosts allows the photochromic behavior of Ph-DHA to be examined across a wide range of polymer microenvironments under comparable preparation and irradiation conditions. Such a comparison is important because the absence of a simple correlation with one individual polymer parameter does not necessarily indicate that the matrix has no effect. Instead, the observed kinetics may reflect a combined contribution of several matrix-related factors, including local polarity, free volume, and structural relaxation of the host. The following sections therefore analyze both the spectral changes induced by UV irradiation (time-dependent coloration) and recovery kinetics of Ph-DHA/polymer films.

### 3.2 Transmission Spectra Show Photochromic Behavior of Polymer-Ph-DHA Composites

The photochromic transformation of Ph-DHA embedded in polymer matrices is directly reflected in the evolution of the optical transmission spectra of the films. Figure 2 shows representative spectra for Ph-DHA incorporated into PS and PVB, selected as examples of chemically different host environments: a nonpolar aromatic hydrocarbon matrix and a more polar hydroxyl-containing polymer, respectively. The spectra for PVB were recorded before UV irradiation, after continuous exposure to 365 nm light, and after subsequent storage in the dark at room temperature, thereby illustrating both photoinduced coloration and delayed recovery in the ambient conditions. The corresponding transmission spectra for the remaining polymer/Ph-DHA composites are presented in Figure S2 in the Supporting Information Materials, enabling comparison of the steady-state spectral response across the full set of investigated polymer matrices.

**Figure 2:**
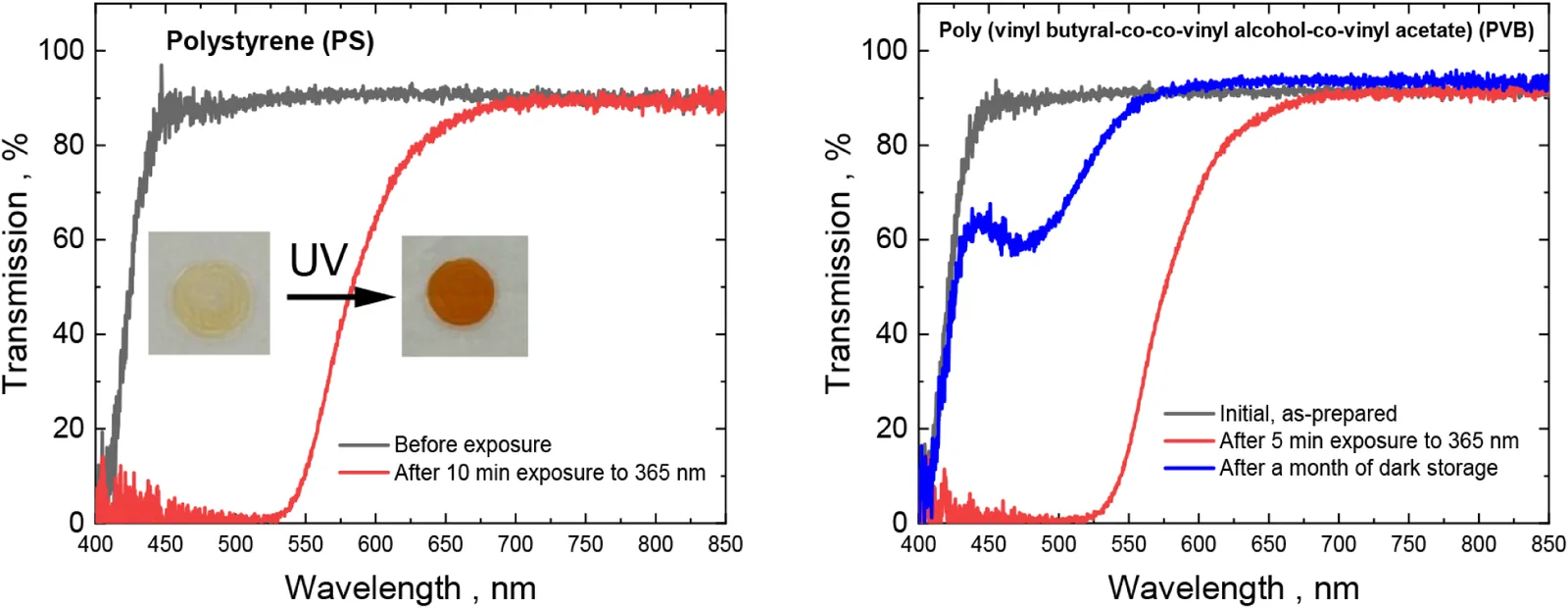
Optical transmission of selected polymer matrices with embedded Ph-DHA before and after exposure with 365 nm LED, and after a month of storage in the dark at room temperature.

Before UV exposure, all polymer/Ph-DHA films exhibited a pronounced absorption edge in the near-UV/blue spectral region. The apparent optical onset energies estimated from the transmission spectra were clustered within a relatively narrow range of approximately 2.7–2.9 eV. This similarity indicates that the ground-state optical response of Ph-DHA is only weakly affected by the chemical nature of the polymer host. Thus, despite differences in polarity, chemical functionality, aromatic content, and segmental mobility, Ph-DHA retains comparable initial optical characteristics in the investigated solid matrices.

Continuous irradiation with 365 nm light induced pronounced changes in the transmission spectra of all films. After approximately 5–10 min of UV exposure, the absorption edge shifted substantially toward longer wavelengths, and the apparent optical onset energies decreased to approximately 1.8–1.9 eV. This corresponds to an overall photoinduced change of about 0.9–1.0 eV and is consistent with the formation of the open VHF form, whose extended π-conjugation leads to enhanced absorption in the visible spectral region. The spectral shift was accompanied by a clear visual coloration of the films, as illustrated for the PS-based composite in Figure 2.

The UV-induced optical changes were not permanent and gradually decayed during storage in the dark at room temperature. The spectrum of the PVB-based film recorded after one month of dark storage demonstrates partial recovery of the initial transmission profile, confirming the thermally reversible character of the DHA/VHF transformation in the solid polymer environment. At the same time, the recovery was incomplete within this period in the spectral region where the greatest photoinduced changes were observed, indicating delayed relaxation of the photoinduced state in the solid matrix.

Importantly, neither the initial nor the photoinduced apparent onset energies showed a clear systematic dependence on a single polymer descriptor, such as polarity, hydrogen-bonding capability, or backbone structure. In addition, the transmission level in the transparent spectral region remained nearly unchanged before and after UV exposure, suggesting that the observed spectral evolution is dominated by molecular photoisomerization rather than by scattering, film degradation, or large-scale morphological changes. Therefore, the transmission spectra confirm that Ph-DHA preserves pronounced photochromic activity across chemically diverse solid polymer matrices, while the steady-state spectral positions of the initial and photoinduced forms remain broadly similar for all hosts.

These results indicate that the fundamental DHA-to-VHF phototransformation is retained in a solid state independently of the selected polymer matrix. However, the similarity of the steady-state spectral shifts does not exclude matrix-dependent effects on the rate of coloration or recovery. For this reason, the time-dependent evolution of the optical response was further analyzed by in situ transmission measurements across the absorption-edge region, as discussed in the following section.

### 3.3 Photoinduced Changes in Optical Transmission

The in situ kinetic curves simultaneously recorded at different wavelengths under continuous UV exposure with 365 nm LED are presented in Figure 3 for PS and PVB polymer matrices as typical examples (data for other polymers are shown in Figure S3 of Supporting Information). In most polymers, the produced photochromism was quasi-stable in ambient conditions for at least a day after the LED was turned off (the first 5 or 10 min after the exposure are shown in Figure 3 or Figure S3). It is consistent with the extended (half lifetime constants ∼ 3–9 days) recovering kinetics observed at room temperature in DHA embedded into PMMA.^21^

**Figure 3:**
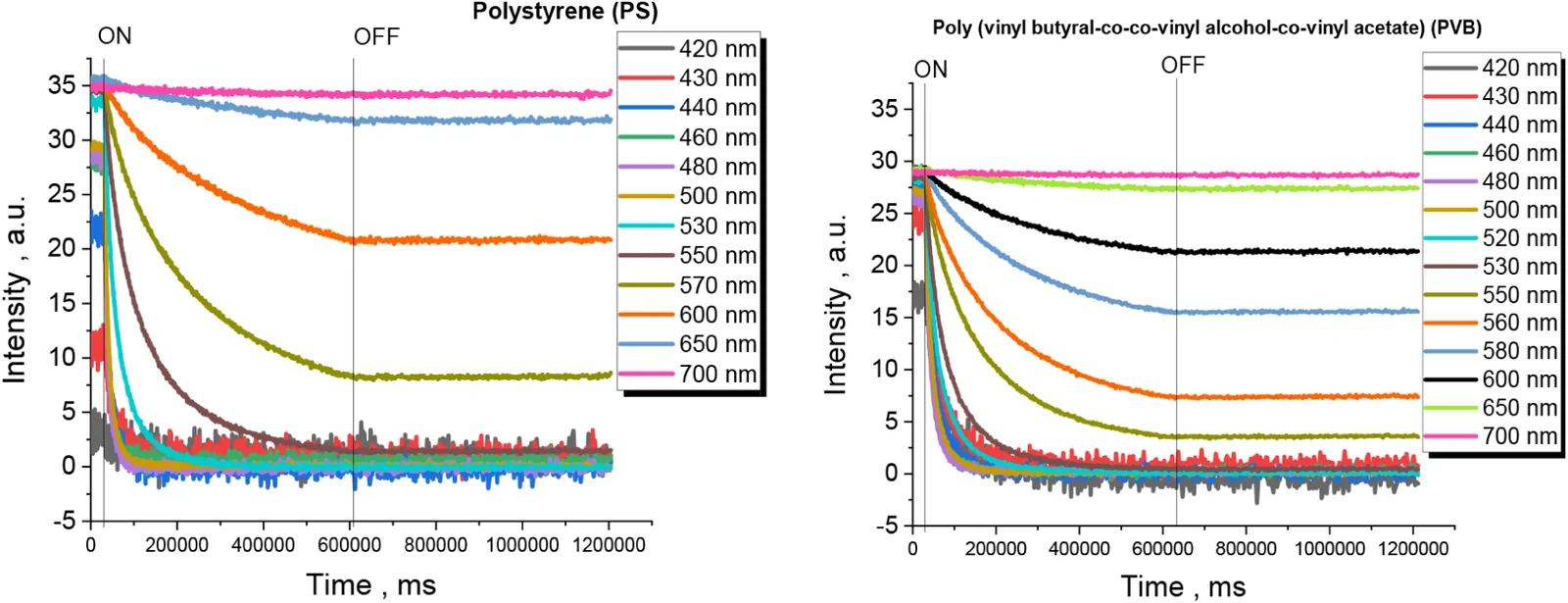
Typical time dependences of the integrated transmitted intensity recorded simultaneously at the indicated wavelengths for the selected Ph-DHA samples. ON and OFF indicate the beginning and the end of exposure with 365 nm LED.

The smoothed in situ absorbance kinetics (calculated from transmittance as *A* = ln 100/*T*) are shown in Figure 4 and Figure S4 for all investigated polymer films under continuous UV exposure. It can be noticed that the kinetics recorded at wavelengths smaller than 530 nm are saturated (or close to saturation in some samples), whereas kinetics recorded at longer wavelengths are mostly incomplete (unsaturated).

**Figure 4:**
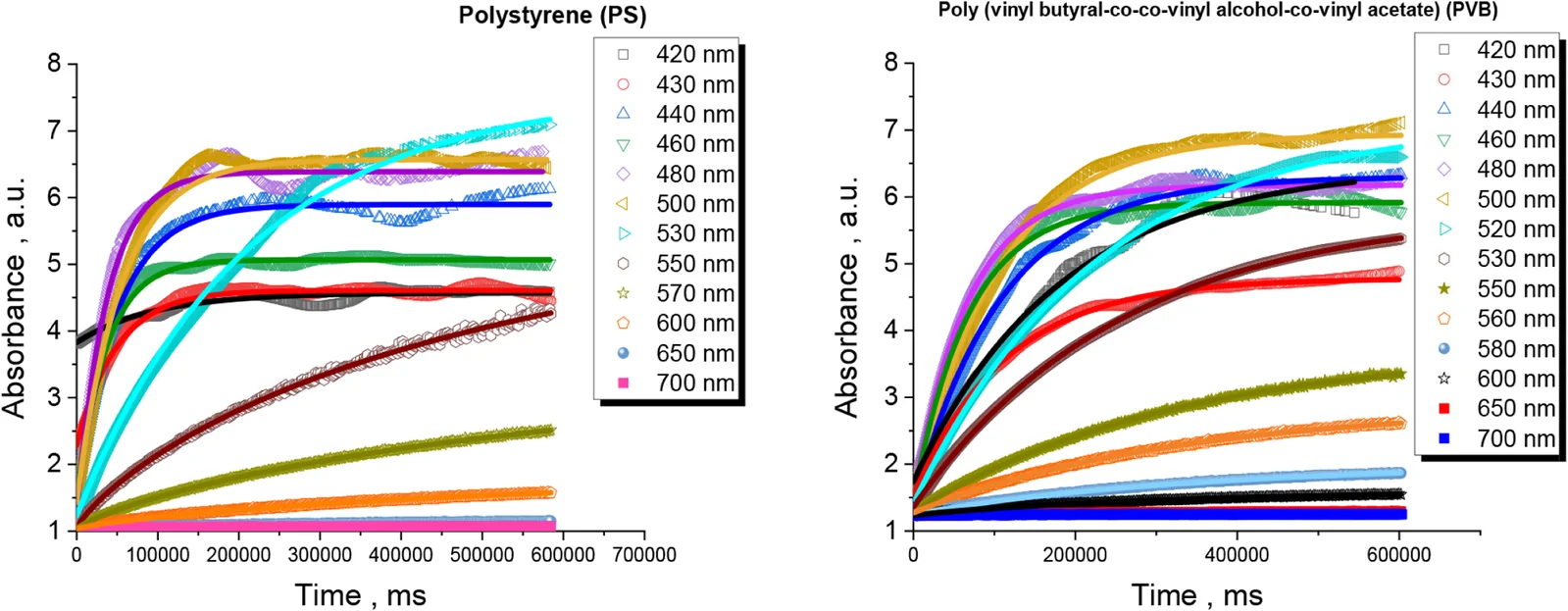
Absorbance kinetic curves recorded at indicated wavelengths for Ph-DHA in the selected polymer matrices under continuous UV exposure with 365 nm LED. Solid lines represent fits to function (5).

In monophotonic systems with negligible thermal relaxation during irradiation, the rate of photochemical reaction is proportional to the concentration (*N)* of the photoactive species, and the photochemical reaction under monochromatic irradiation in a well-stirred fluid solution is usually given by a first-order rate equation:^25–27^

1 $$\frac{dN}{dt}=-k_{app}N$$

where *k*_*app*_ depends on many parameters, such as quantum yield, molar extinction coefficients of photoreactive and photo-inert species, photokinetic factor, incident light flux, and optical pathlength.

Equation 1 yields exponential-like families of solutions (or linear in the extreme case for high molar extinction coefficients) for the time dependence of absorbance (*A*), which is assumed to be directly proportional to the concentration of the photoreactive species (*N*) in the solution, as described in the extensive literature on photochromism.^25–27^

In the case under consideration, we have a monophotonic system (Ph-DHA embedded into a photo-inert polymer), but in a disordered solid state, not in a fluid solution. In condensed media, photochemical transformations are governed by a pair of coupled partial differential equations:^27,28^

2 $$-\frac{\partial N\left(z,t\right)}{\partial t}=kIF\left(N\right)$$

3 $$-\frac{\partial I\left(z,t\right)}{\partial z}=\left(\alpha_{N}N+\alpha_{P}P+\alpha_{M}M\right)I$$

Here, *P* and *M* denote concentrations of product and inert medium; *α*_*i*_ are the respective molar extinction coefficients; *t* and *z* refer to time and depth below the film surface; *I*(*z,t*) represents the light intensity at a depth *z* at time *t*; *F*(*N*) is a rate function dependent on the system.

The nonlinear coupling of (2) and (3), and difficult (or impossible) conventional chemical analysis used to obtain product and kinetic information in disordered solids, creates a lot of complications for the exact theoretical solutions, if they can be obtained at all. In solid polymer matrices, the photoactive molecules are located in spatially heterogeneous and dynamically constrained environments. Therefore, the observed kinetics can include contributions not only from the intrinsic DHA-to-VHF photochemical transformation, but also from local packing, free volume, polymer segmental mobility, and depth-dependent light absorption. As a result, the measured photo response is expected to represent an ensemble-averaged solid-state process rather than a single uniform first-order reaction. Various approximate methods of dealing with the nonlinear coupling between (2) and (3) have been discussed in the literature,^28^ but in the case of disordered solid polymer matrices, the universal theoretical law governing the optical changes has not been proposed yet.

Owing to the micrometer-scale thickness of the prepared polymer films and the limited penetration depth of 365 nm UV light, the photoinduced changes are expected to be most pronounced in the irradiated near-surface region. Because the films were prepared under comparable conditions and had similar thicknesses, variations in film thickness are unlikely to be the dominant factor controlling the differences between the photochromism in various polymer matrices, especially in the wavelength range where the transmission changes approach saturation (Figures 2, 3).

On the other hand, more spatially constrained structural dynamics should result in a much broader distribution of reaction times—scenario which usually is described empirically by a stretched exponential (Kohlrausch’s) law:^29,30^

4 $$\delta \left(t\right)=\delta_{0}\exp\left\{-{\left(\int_{0}^{t}\frac{dt}{\tau }\right)}^{\beta }\right\}$$

where *δ*_0_ is the initial departure of physical quantity *δ* (*t*) from equilibrium; *τ* is the effective time constant; and dimensionless stretching exponent *β* is a so-called fractional factor (also known as non-exponentionality or dispersivity index), 0 < *β* ≤ 1 (*β* =1 for single-exponential relaxation processes and smaller for dispersive processes).

This law is experimentally observed for time evolution of many physical quantities (including photoinduced effects) in various disordered electronic and molecular systems,^29–32^ such as the considered polymer matrices. For this reason, the observed changes in the optical absorbance Δ*A*(*t*) were fitted with stretched exponential function assuming *τ* is not dependent on time:

5 $$\Delta A\left(t\right)=C_{1}-C_{2}\times e^{-{\left(\frac{t}{\tau }\right)}^{\beta }}$$

where *C*_1_ and *C*_2_ are fitting constants.

The unrestricted fits to (5) converged for all kinetics recorded under continuous UV exposure (Figure 4, Figure S4) with dispersivity index *β* varying with wavelengths within 0.9–1.0 range for PVAc, PVB and PSIS polymers, and 0.8–1.0 for PEMA, PVK, PVP, P4VP, and PS polymer matrices (Table 1). The variation 0.7–0.9 was obtained for PEPC oligomer (Table 1). According to our observations, *β* parameter deviates more from unity toward shorter and especially longer wavelengths (Table 1). The close proximity of dispersivity index to unity for the wavelengths within 460–520 nm range means that influence of the host matrix on the non-exponentiality of Ph-DHA photochromism is minimal for the transformations with maximum rate, and the first-order kinetics could still be a good approximation for most practical cases. As such, the fits to (5) with a restriction of *β* = 1.0 (single exponential) was performed and the obtained time constants of the reaction are plotted in Figure 5 for each polymer as a function of monitored wavelength for comparison. Despite the *β* = 1.0 restriction, the fits converged well for all photoinduced in situ kinetics at all wavelengths. From unrestricted and restricted fits, it can be concluded that the fastest changes (smallest *τ* values) occur within the optical absorption edge region, where the main absorption band of VHF is usually located (460–500 nm).^12,24,33^ At longer wavelengths, the process becomes significantly slower, and for PSIS polymer time dependence even splits into two exponential components for the kinetics recorded at the wavelengths longer than 530 nm (Figure S4, Table 1) with only*τ* for the faster component shown in Figure 5. In the most general case, the time constant would depend on many factors, including UV light flux, UV wavelength, temperature, coupling function, etc.

**Table 1:** Dispersivity Index (±0.05) and Time Constants (±5%) of In Situ UV-Induced Photodarkening Obtained from Unrestricted Fits to eq 5 for the Kinetics at Selected Wavelengths

	Wavelength (nm)									
	430		460		480		500		520	
Polymer	β	τ, s	β	τ, s	β	τ, s	β	τ, s	β	τ, s
**PS**	1	48.9	1	36.9	1	37.8	1	54.8		
**PVB**	0.89	115.9	1	69.3	1	67.8	1	106.3	1	224.6
**PVK**	0.83	80.8	0.89	67.1	0.86	56.5	0.86	64.3	0.87	75.6
**PVAc**	0.94	110.8	1	61.8	1	83.7	0.95	94.4	0.92	138.0
**PVP**	1	224.4	1	122.2	1	71.7	1	106.9	0.92	149.1
**PEPC**	0.7	592.8	0.79	198.5	0.9	152.5	0.83	138.1	0.77	244.7
**PSIS**	1	77.1	1	61.4	1	57.8	1	84.5	0.98	190.4
**P4VP**			1	79.1	1	68.1	1	94.6	1	99.5
**PEMA**	0.93	108.9	1	78.9	1	73.3	0.94	110.1		

	Wavelength (nm)							
	530		550		580		600	
Polymer	β	τ, s	β	τ, s	β	τ, s	β	τ, s
**PS**	1	211.7	0.82	522.7	0.81	69.9	0.79	80.0
**PVB**	1	255.1	0.91	371.2	0.91	384.1	0.86	422.3
**PVK**	088	86.3	0.84	102.0	0.86	107.9	0.82	110.2
**PVAc**	0.92	145.7	0.9	179.1	0.88	201.6	0.84	191.4
**PVP**	0.83	296.7	0.79	612.5	0.76	1181.2	0.82	591.5
**PEPC**	0.76	238.5	0.81	239.3	0.76	414.5	0.75	453.9
**PSIS**	0.92, 1	260.7, 216.7	0.87, 1	2112.6, 310.2	1, 1	3.5 × 10^5^, 615.3	1, 1	1.7 × 10^5^, 559.6
**P4VP**	1	133.1	0.82	423.9			0.74	1228.9
**PEMA**	0.9	163.8			0.81	281.5	0.76	266.7

**Figure 5:**
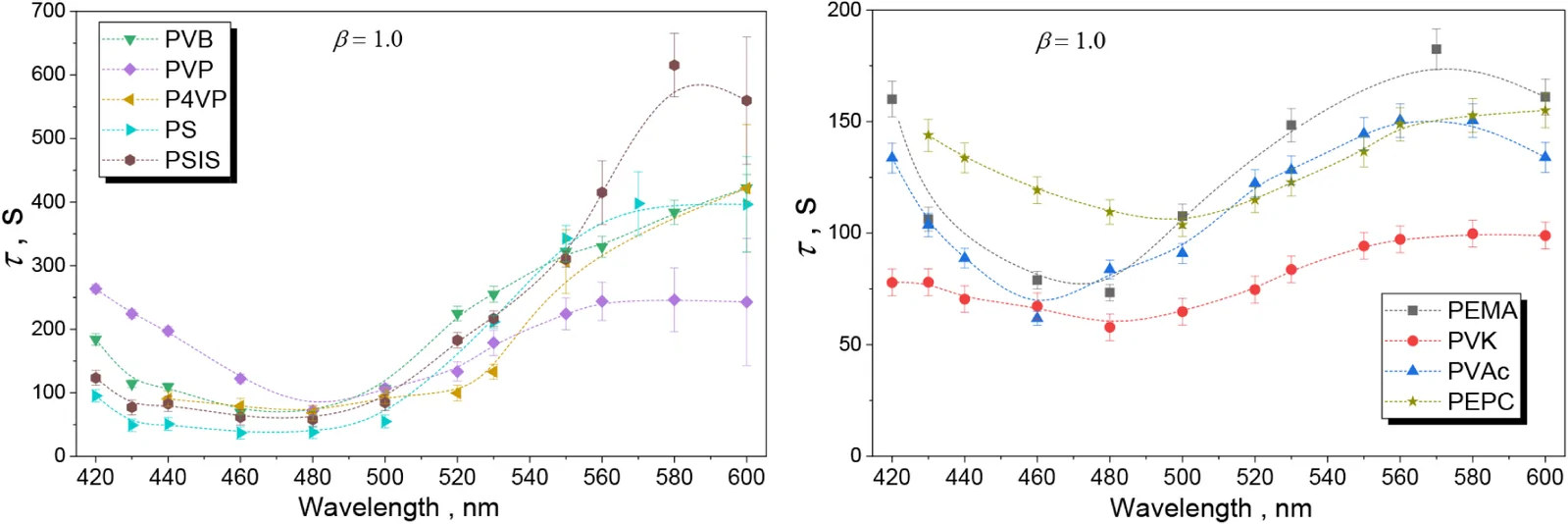
Wavelength dependence of the time constants for in situ photoinduced changes as obtained from fitting to function (5) and *β* = 1.0 constraint.

Despite a significant difference of *τ* for different polymer hosts, our data shows no clear dependence on the nature of the polymer matrix if only one parameter (such as polarity, molecular weight, or polarizability) is considered at a time.

Thus, the absence of a simple one-parameter correlation does not imply that the polymer matrix is passive. Rather, it suggests that the photoinduced coloration of Ph-DHA in solid films is governed by a combination of local matrix effects, including polarity, free volume, segmental mobility, packing density, and weak chromophore-polymer interactions. Different polymer hosts may therefore produce broadly similar steady-state spectral shifts while still providing different microscopic environments for the DHA-to-VHF transformation. The kinetics observed here should consequently be considered as an average response of Ph-DHA molecules distributed over a broad range of local polymer environments, which is the reason for deviation of dispersivity index from unity in the unrestricted fits to (5), and significant difference in *τ* values.

### 3.4 Recovery Kinetics

The reversibility of the Ph-DHA/VHF transformation in polymer matrices was evaluated by monitoring the recovery of the optical transmission after UV irradiation. At room temperature, the photoinduced state was relatively long-lived: the optical properties of the films were almost fully restored only after prolonged storage in the dark, as illustrated for the PVB-based film in Figure 2. Heating strongly accelerated the recovery process. On a laboratory time scale, substantial restoration of transparency was observed at temperatures of 80 °C and above, with faster recovery at higher annealing temperatures.

Thermal recovery of transmission shows a noticeable wavelength dispersion for some polymers, as shown in Figure 6 and Figure S5.

**Figure 6:**
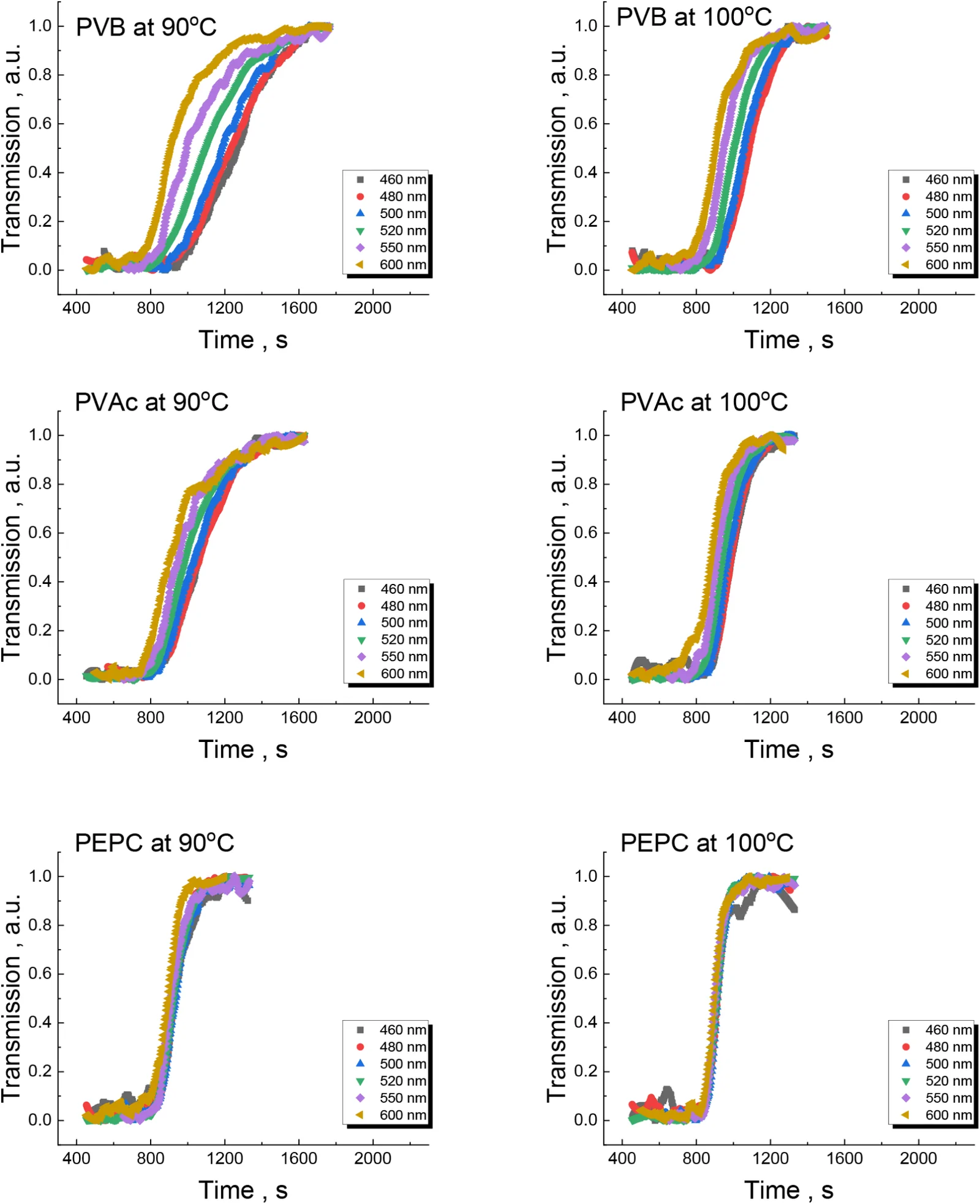
Comparison of recovery kinetics recorded on different wavelengths at 90 °C (left panel) and 100 °C (right panel) for typical polymers with high, medium and low dispersion of thermal recovery process.

The normalized transmission recovery first is noticed at longer wavelengths, where the smallest (and unsaturated) photoinduced darkening effect is observed. The photoinduced changes at 460–500 nm, where the maximum photochromic response was recorded, recover more slowly and show a noticeable delay compared with the 550–600 nm kinetics for some samples (Figure 6 and Figure S5). This dispersion decreases with the increase in the annealing temperature and vanishes for the PEPC oligomer. The dispersion is even more pronounced at room temperature, as can be concluded from the analysis of PVB transmission spectra in Figure 2, where transmission is fully recovered after a month of dark storage for the 550 nm and higher wavelengths, but still shows a noticeable effect for the wavelength range of maximum photoinduced changes (around 480–500 nm), indicating delayed kinetics. The wavelength-dependent recovery further supports the idea that the photoinduced state in solid films is not represented by a single uniform population. The delayed recovery in the 460–500 nm region, where the largest photoinduced absorption changes are observed, may reflect a subset of VHF species stabilized more efficiently by local polymer environments. These environments can differ in polarity, packing density, free volume, or proximity to specific functional groups, depending on the host matrix. Thus, the polymer matrix affects the recovery not through a single dominant macroscopic descriptor, but through the distribution of microscopic sites available to the embedded photochrome.

In contrast to the UV-induced coloration kinetics, the reverse VHF-to-DHA process was described by a stretched-exponential function with greater variation of *β* within ∼0.6–1.0 (single exponential) range when changes in absorbance are fitted to

6 $$\Delta A\left(t\right)=Const\times e^{-{\left(\frac{t}{\tau }\right)}^{\beta }}$$

Examples of the unconstrained fits to the stretched exponential relaxation kinetic function (6) are shown in Figure 7 for two cases when stretch exponent *β* was close to 1 (exponential) and the other typical case when it was close to 0.6. The latter exponent is predicted by Phillips axiomatic diffusion-to-traps theory when all relaxation channels are equally activated in 3-dimensional space.^34^ Stretch exponent *β* = 0.60 ± 0.05 was obtained for PEMA, PVK and PVP polymer matrices for both annealing temperatures 90 °C and 100 °C, whereas it demonstrated a change from 0.6 at 90 °C to 1.0 at 100 °C annealing for P4VP and PSIS polymers (Figure S6). The tendency to overall *β* increase as the temperature increased from 90 °C to 100 °C is similar to simple inorganic polymers like chalcogenide glasses, where *β* increased for structural relaxation approaching glass transition temperature *T*_*g*_.^35^ Therefore, distance from *T*_*g*_ and possible superposition with structural relaxation of disordered polymer matrix should be additionally considered when thermal recovery is studied. The relaxation time constant *τ* as obtained from unrestricted fitting to (6), demonstrates a noticeable drop for most samples when annealing temperature increases from 90 °C to 100 °C (Figure S6), except the PSIS polymer where change in *β* from 0.6 to 1.0 causes the inverse temperature dependence for *τ* (Figure S6). However, no obvious dependence of *β* or *τ* on the molecular weight or polarity was observed in our experiments.

**Figure 7:**
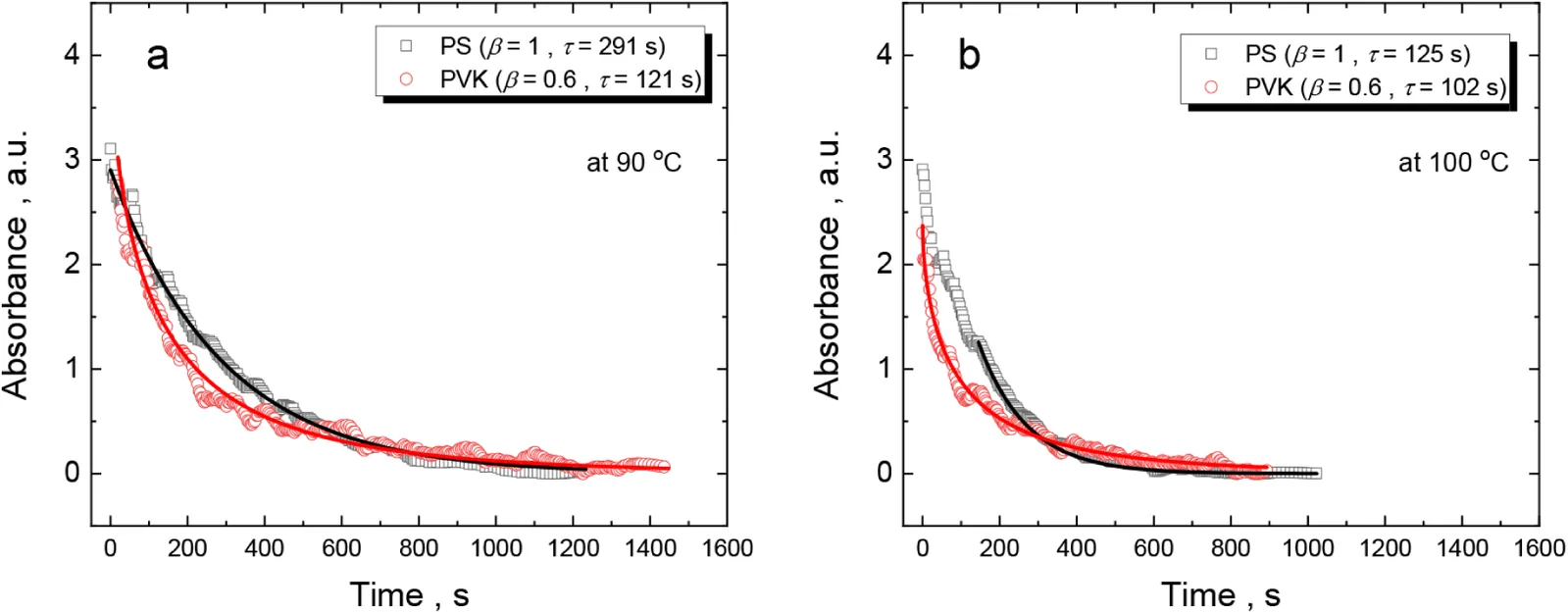
Comparison of recovery kinetics recorded on a 500 nm wavelength at 90 °C (a) and 100 °C (b) for two selected polymers (examples) fitted to a stretched exponential relaxation function (6) with different *β*.

The deviation of stretching exponents from unity suggests that thermal recovery is not governed solely by the intrinsic VHF-to-DHA ring-closure barrier, but also by the distribution of local relaxation environments provided by the host polymer. In rigid or heterogeneous matrices, different Ph-DHA molecules may experience different degrees of conformational restriction and different local free-volume conditions, leading to a distribution of recovery times. Conversely, more dynamically permissive local environments may facilitate molecular reorganization required for ring closure and thus produce kinetics closer to a simple exponential response. Therefore, the stretched-exponential behavior provides indirect evidence that polymer segmental relaxation and local packing contribute to the thermal recovery of the photoinduced state in some polymers.

## 4 Conclusions

Ph-DHA was successfully embedded into nine optically transparent solid polymer matrices, and pronounced photochromism was observed in all films investigated. Continuous 365 nm irradiation induced a substantial red shift of the apparent absorption edge, consistent with formation of the open VHF form in the solid state. The photoinduced changes in absorbance followed a stretched exponential time dependence over the main part of the coloration process, approaching exponential first order kinetics for the fastest optical response within 460–520 nm spectral region, where the contribution from VHF-related absorption is most pronounced. So, exponential first-order kinetics is still a good approximation for the photochromism of Ph-DHA embedded into optically inert polymer matrix.

The comparison of different polymer hosts showed that the coloration kinetics cannot be correlated with a single macroscopic polymer descriptor, such as polarity, molecular weight, aromaticity, or chemical functionality. Instead, the results point to an averaged response of Ph-DHA molecules distributed over different local microenvironments within the solid films. Polymer free volume, segmental mobility, local packing, and weak chromophore-host interactions are therefore likely to act together in defining the apparent photochromic kinetics.

The initial optical properties of the films were recovered either slowly during dark storage at room temperature or more rapidly upon thermal annealing above 80 °C. The thermal recovery kinetics were well described by a stretched-exponential function with stretching exponent *β* ranging from 0.6 to 1.0. This behavior indicates that the VHF-to-DHA recovery in solid films to some degree is coupled to a distribution of relaxation pathways in polymer matrices. The wavelength-dependent recovery, especially the delayed relaxation in the 460–500 nm region, further supports the presence of differently stabilized photoinduced species in the polymer environment.

Overall, the study demonstrates that Ph-DHA retains robust and thermally reversible photochromism across chemically diverse polymer matrices. At the same time, the polymer host controls the apparent coloration and recovery kinetics through a complex distribution of local environments. These findings provide a useful phenomenological basis for the design of DHA-based photoresponsive polymer films, where the matrix should be considered an active component of the photochromic system rather than an inert support.

## Supplementary Material



## Notes

Figures S1–S6 with the data for all nine polymers (PDF)

## Abbreviations

Ph-DHA: phenyl-substituted dihydroazulene derivative
VHF: vinylheptafulvene
PS: polystyrene
PSIS: polystyrene-block-polyisoprene-block-polystyrene
PEMA: poly­(ethyl methacrylate)
PVAc: poly­(vinyl acetate)
PVB: poly­(vinyl butyral-co-vinyl alcohol-co-vinyl acetate)
PVP: polyvinylpyrrolidone (PVP), poly­(4-vinylpyridine)
PVK: poly­(N-vinylcarbazole)
PEPC: oligomer poly­(epoxypropyl-carbazole)
PMMA: poly­(methyl methacrylate)
NMR: nuclear magnetic resonance
UV: ultraviolet
LED: light emitting diode
