# Supplementary material for: Photochromism of Dihydroazulene Embedded in Solid Polymer Matrices
Source: J Phys Chem B. 2026 Sep 10;130(38):10076–86. doi: 10.1021/acs.jpcb.6c04116 (PMC13618032; doi:10.1021/acs.jpcb.6c04116)
Supplement: Supplementary file 1 [file jp6c04116_si_001.pdf]

Photochromism of Dihydroazulene embedded into solid polymer matrices

Roman Golovchak[1*], Liudmila Loghina[2**], Joshua Rye[1], Emmabeth Parrish Vaughn[1], Andriy Kovalskiy[1], Keira Scott[1], Calleway Schmidt[1], Roman Jambor[3], Miroslav Vlcek[2,3]

[1] *Department of Physics, Engineering and Astronomy, Austin Peay State University, Clarksville, TN 37044, USA*

*corresponding author: holovchakr@apsu.edu

[2]*Center of Materials and Nanotechnologies, Faculty of Chemical Technology, University of Pardubice, 530 02 Pardubice, Czech Republic.*

[3]*Department of General and Inorganic Chemistry, Faculty of Chemical Technology, University of Pardubice, 532 10 Pardubice, Czech Republic.*

**corresponding author: liudmila.loghina@upce.cz

Phenyl-substituted dihydroazulene derivative (Ph-DHA) was embedded into nine solid-state polymer matrices commonly used in optoelectronics and photonics: polystyrene (PS), polystyrene-block-polyisoprene-block-polystyrene (PSIS), poly(ethyl methacrylate) (PEMA), poly(vinyl acetate) (PVAc), poly(vinyl butyral-co-vinyl alcohol-co-vinyl acetate) (PVB), polyvinylpyrrolidone (PVP), poly(4-vinylpyridine) (P4VP), poly(N-vinyl carbazole) (PVK), and oligomer poly(epoxypropyl-carbazole) (PEPC).

Figure S1 shows a three-step synthetic sequence used to prepare 2-phenylazulene-1,1(8aH)-dicarbonitrile (Ph-DHA, **3**) compound. In the first step, Knoevenagel condensation of acetophenone with malononitrile afforded 2-(1-phenylethylidene)malononitrile (**1**), which was isolated as a stable crystalline intermediate after chromatographic purification. The subsequent reaction of compound 1 with tropylium tetrafluoroborate under an argon atmosphere at low

temperature produced the corresponding cycloheptatrienyl-substituted intermediate (**2**). Owing to its limited stability, this intermediate was not isolated and was directly used in the next step.

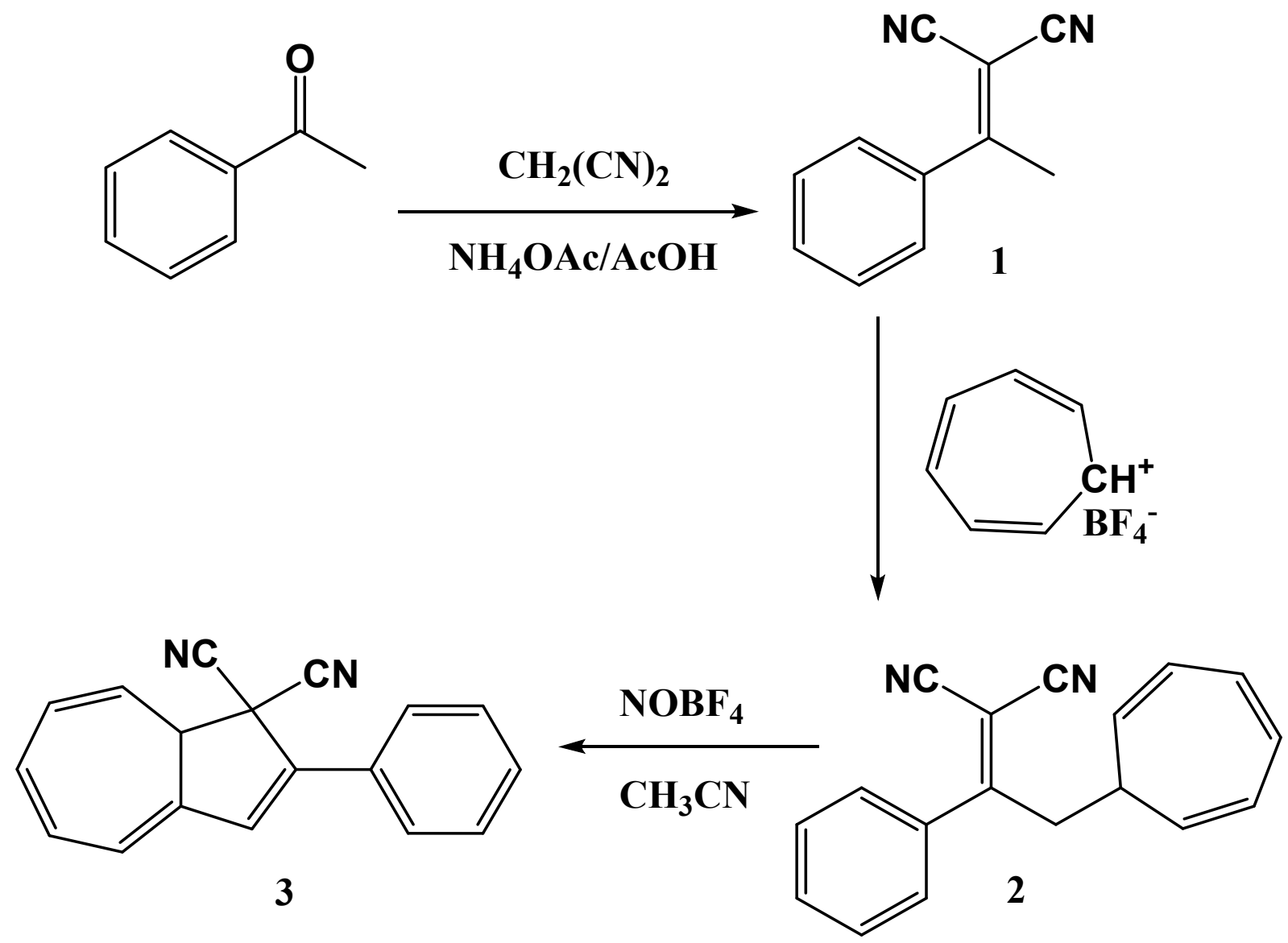

Figure S1. The block-diagram of Ph-DHA synthesis procedure.

The desired DHA framework was formed by treatment of the crude intermediate 2 with nitrosonium tetrafluoroborate in dry acetonitrile under an inert atmosphere and in the absence of light. After chromatographic purification, Ph-DHA was obtained as a stable solid and its structure was confirmed by ^{1}H and ^{13}C NMR spectroscopy.

The Ph-DHA photochromic transformation under continuous UV (365 nm wavelength) radiation results in a pronounced long-wave shift of the optical transmission spectra shown in Figure S2.

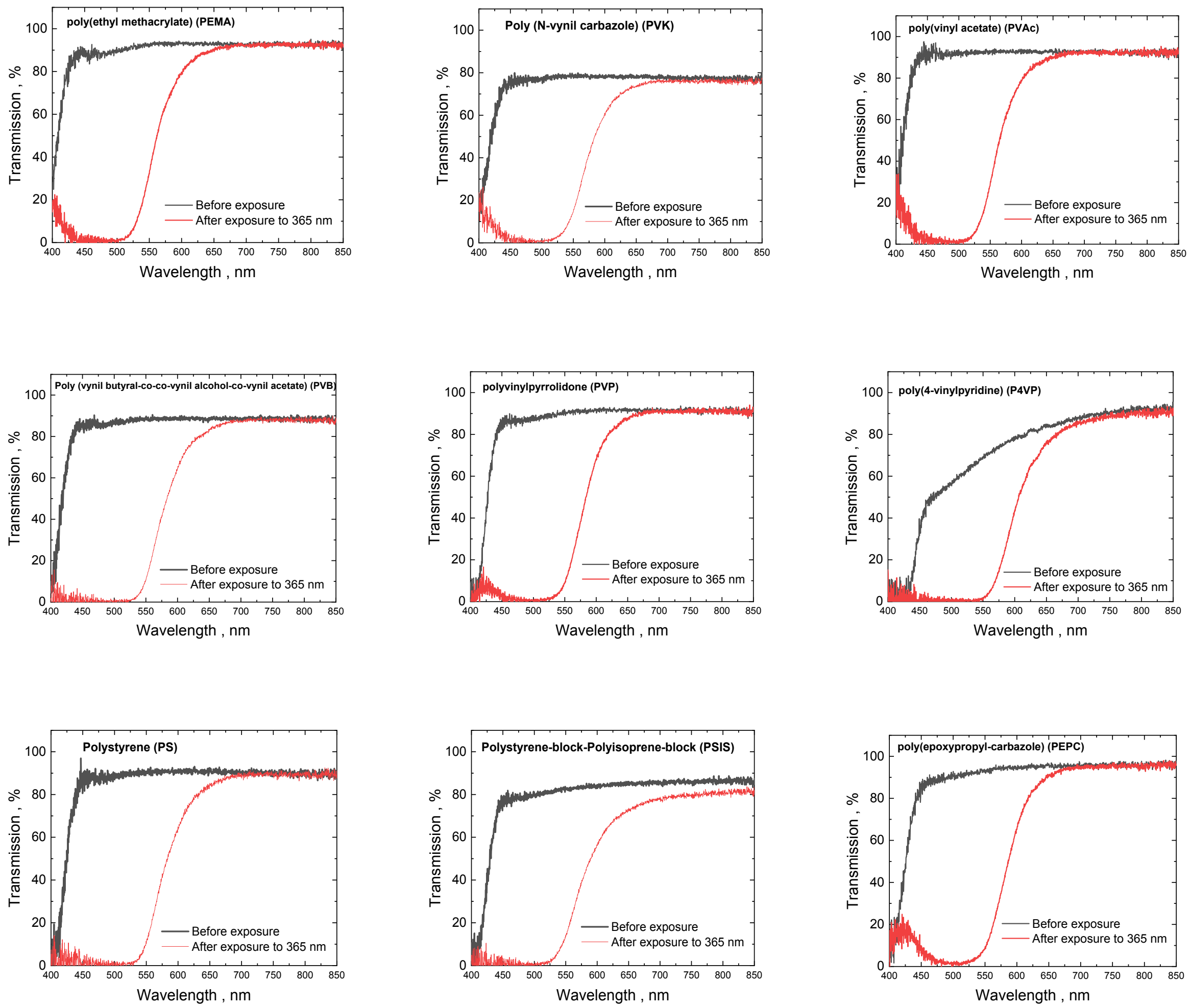

Figure S2. Optical transmission of studied polymer matrices with embedded Ph-DHA before and after 10-min exposure with 365 nm LED. Polymers are arranged according to their molecular weight (highest to smallest).

The *in situ* kinetic transmission curves simultaneously recorded at different wavelengths under continuous UV exposure with 365 nm LED are presented in Figure S3.

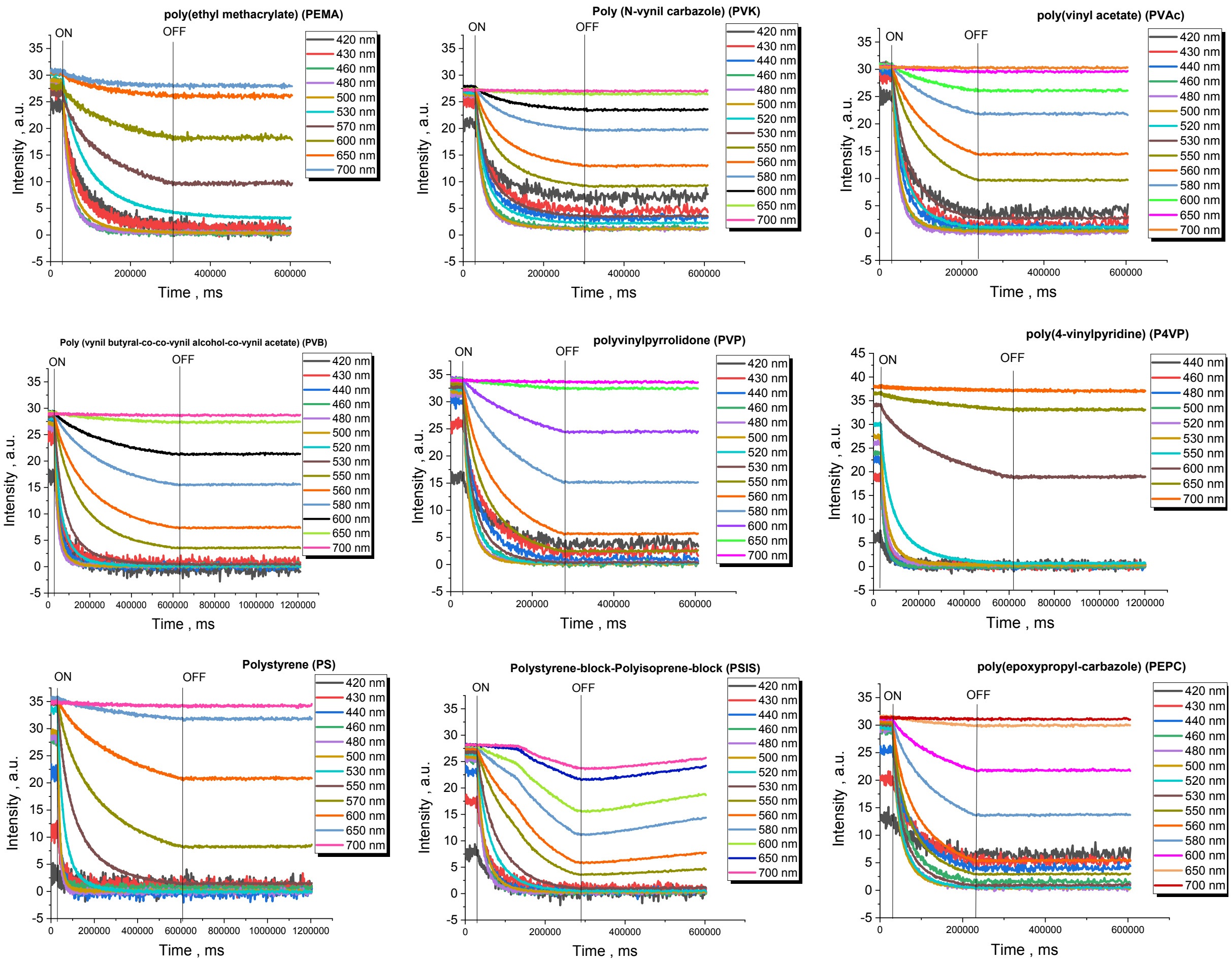

Figure S3. Time dependences of the transmitted intensity recorded simultaneously at the indicated wavelengths for the Ph-DHA samples presented in Figure 1S. ON and OFF indicate the beginning and the end of exposure with 365 nm LED. Polymers are arranged according to their molecular weight (highest to smallest).

The *in situ* absorbance kinetics recalculated from spectra in Fig. S2 as $A = \ln(100/T)$ and their fits to equation (5), are shown in Figure S4 for all investigated polymer films under continuous UV exposure.

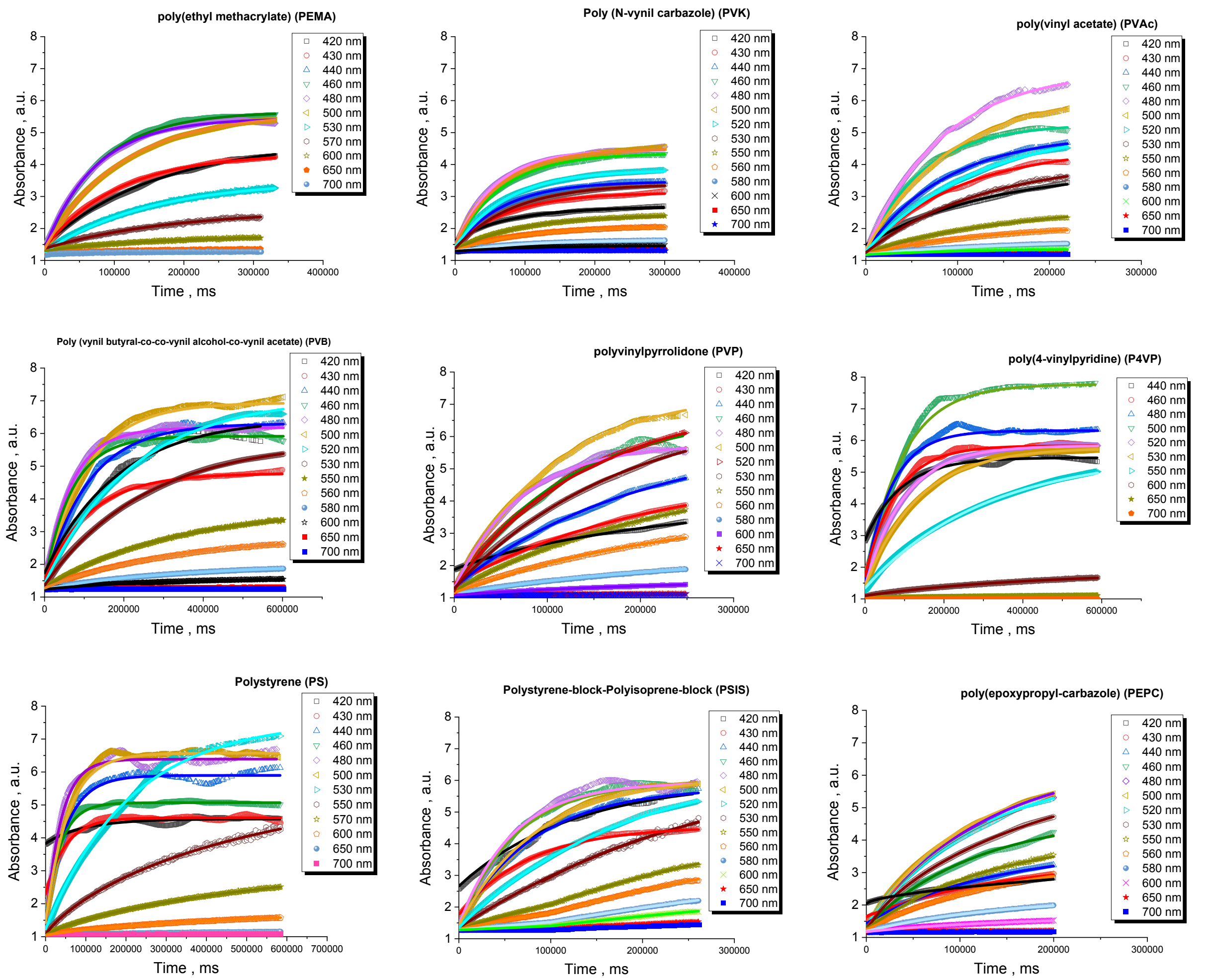

Figure S4. Absorbance kinetic curves recorded at indicated wavelengths for Ph-DHA in studied polymer matrices under continuous UV exposure with 365 nm LED. Solid lines represent fits to function (5) with $\beta = 1$ constraint. Polymers are arranged according to their molecular weight (highest to smallest).

Normalized thermal recovery transmission kinetics recorded in the dark at 90 °C and 100 °C are shown in Figure S5.

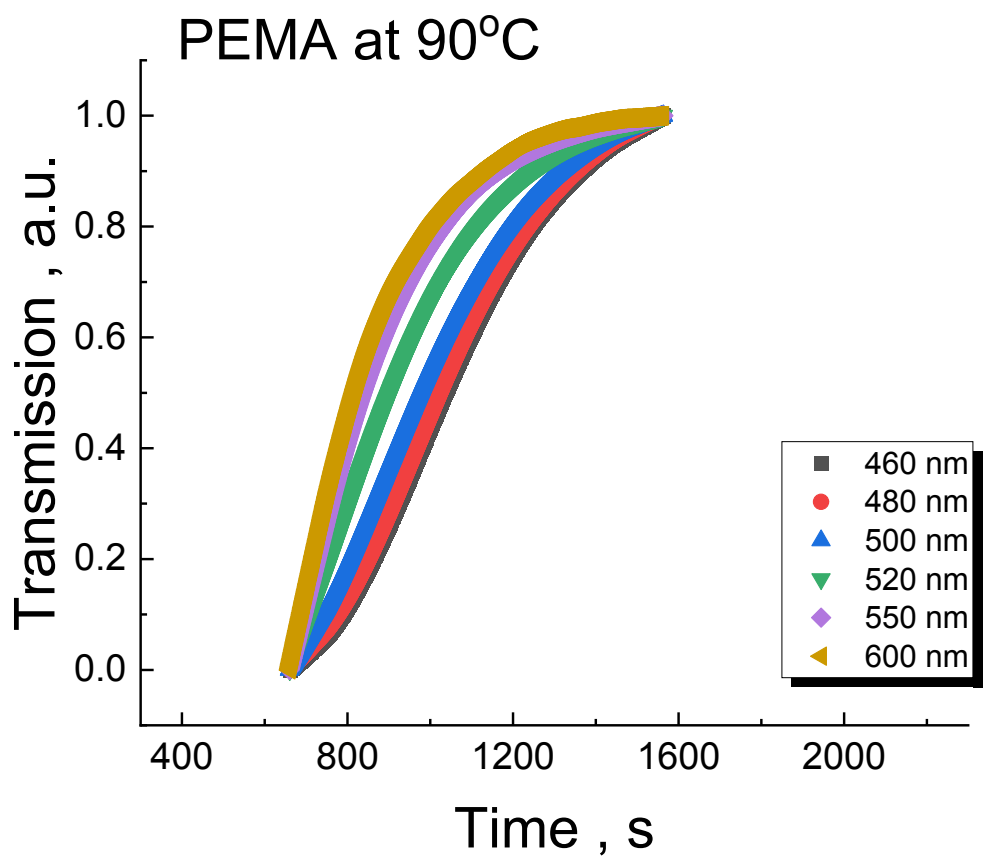
PEMA at 90°C
Transmission , a.u.
0.0
0.2
0.4
0.6
0.8
1.0
Time , s
400
800
1200
1600
2000
460 nm
480 nm
500 nm
520 nm
550 nm
600 nm

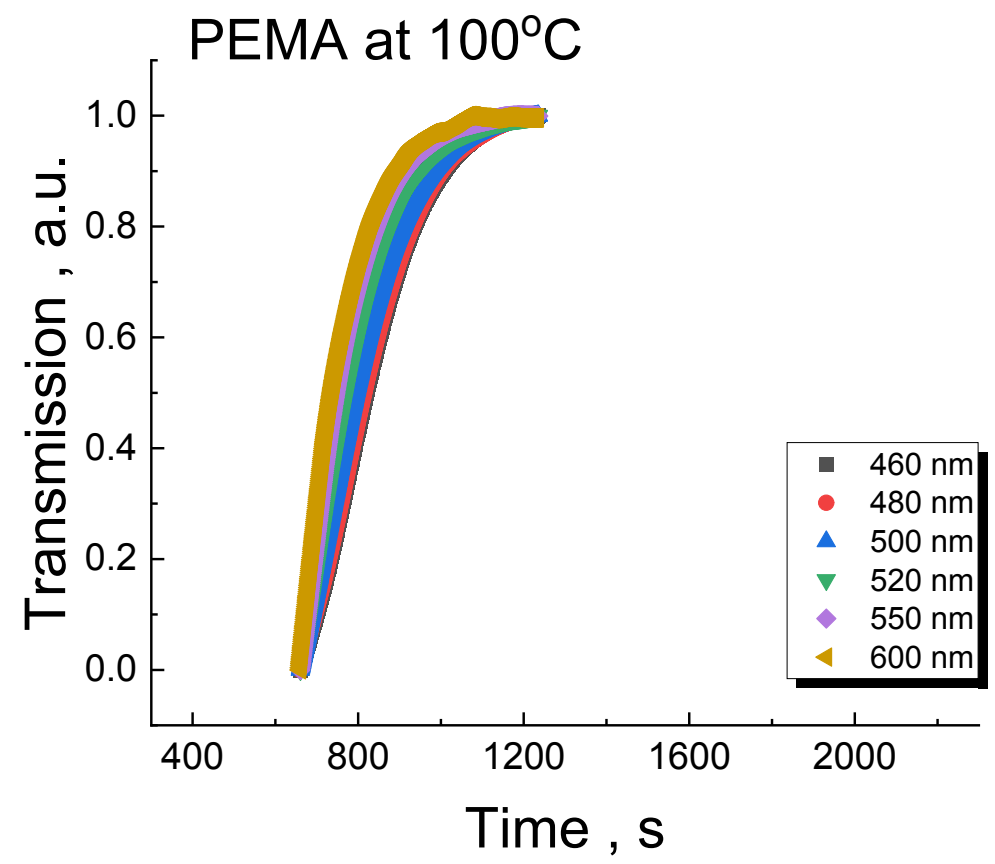
PEMA at 100°C
Transmission , a.u.
0.0
0.2
0.4
0.6
0.8
1.0
Time , s
400
800
1200
1600
2000
460 nm
480 nm
500 nm
520 nm
550 nm
600 nm

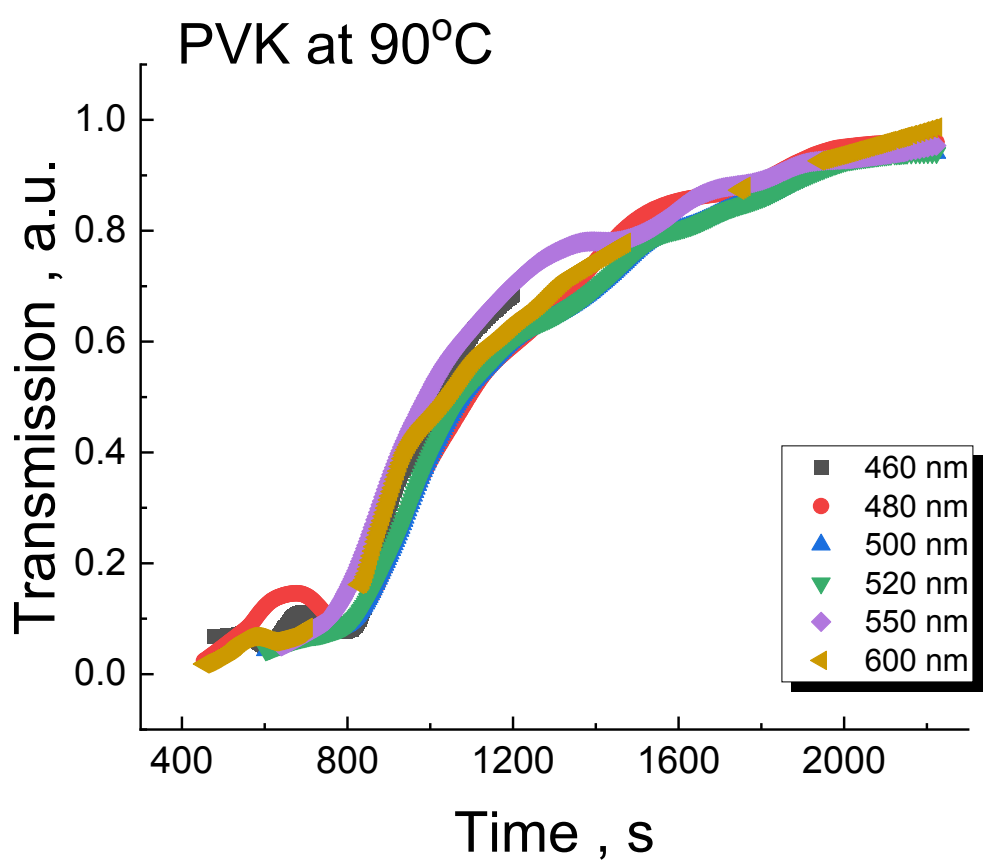
PVK at 90°C
Transmission , a.u.
0.0
0.2
0.4
0.6
0.8
1.0
Time , s
400
800
1200
1600
2000
460 nm
480 nm
500 nm
520 nm
550 nm
600 nm

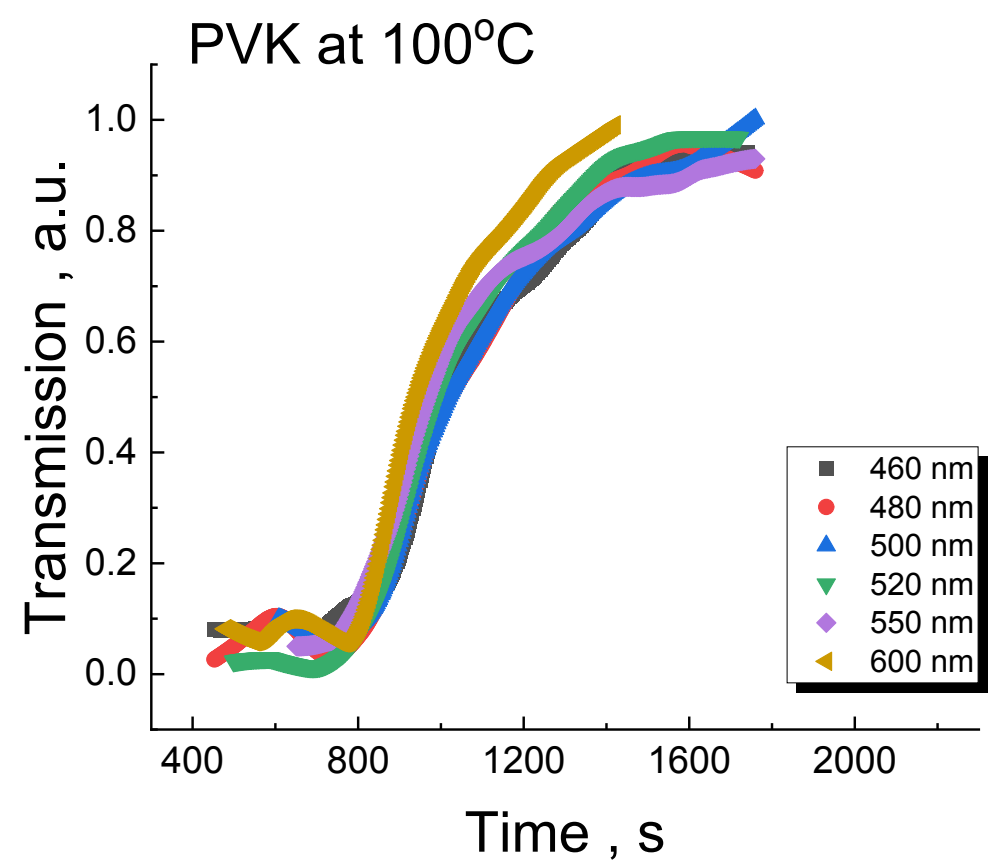
PVK at 100°C
Transmission , a.u.
0.0
0.2
0.4
0.6
0.8
1.0
Time , s
400
800
1200
1600
2000
460 nm
480 nm
500 nm
520 nm
550 nm
600 nm

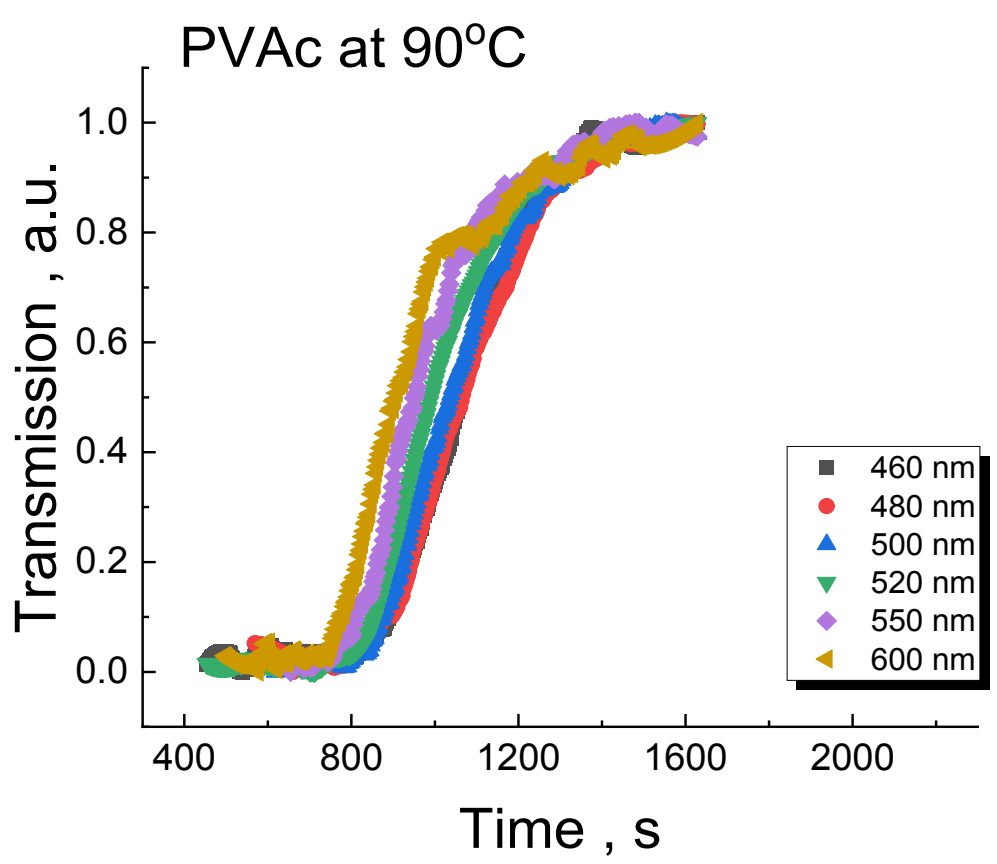
PVAc at 90°C
Transmission , a.u.
0.0
0.2
0.4
0.6
0.8
1.0
Time , s
400
800
1200
1600
2000
460 nm
480 nm
500 nm
520 nm
550 nm
600 nm

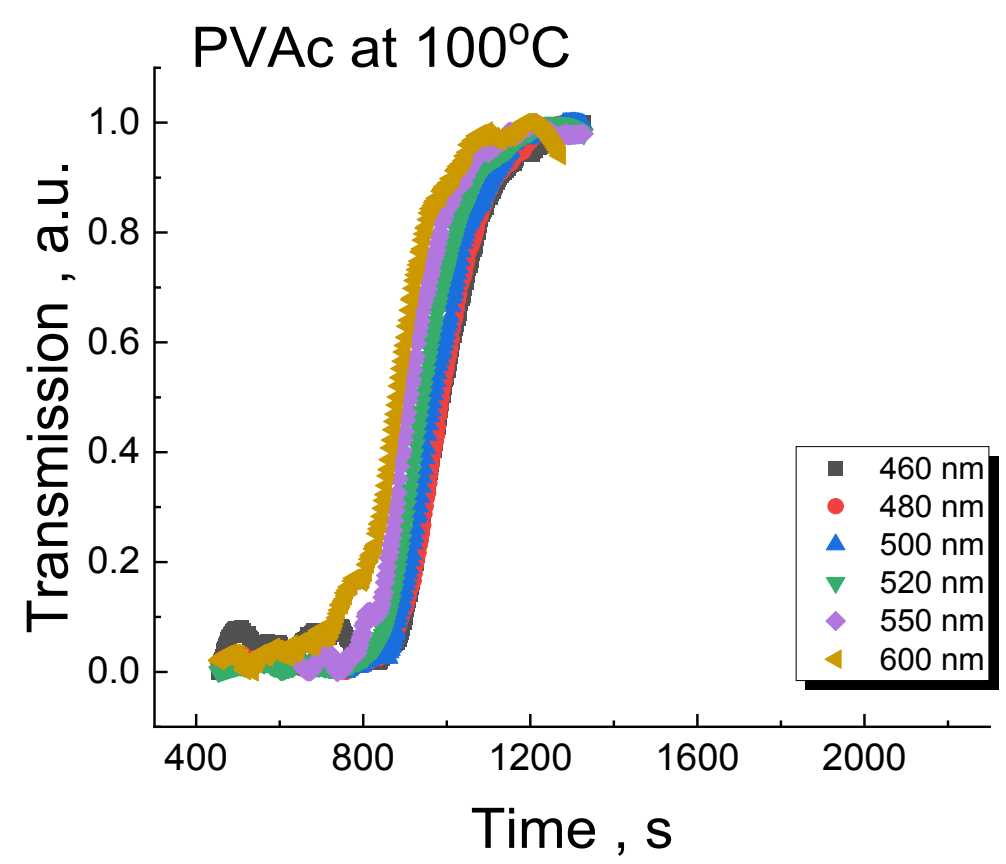
PVAc at 100°C
Transmission , a.u.
0.0
0.2
0.4
0.6
0.8
1.0
Time , s
400
800
1200
1600
2000
460 nm
480 nm
500 nm
520 nm
550 nm
600 nm

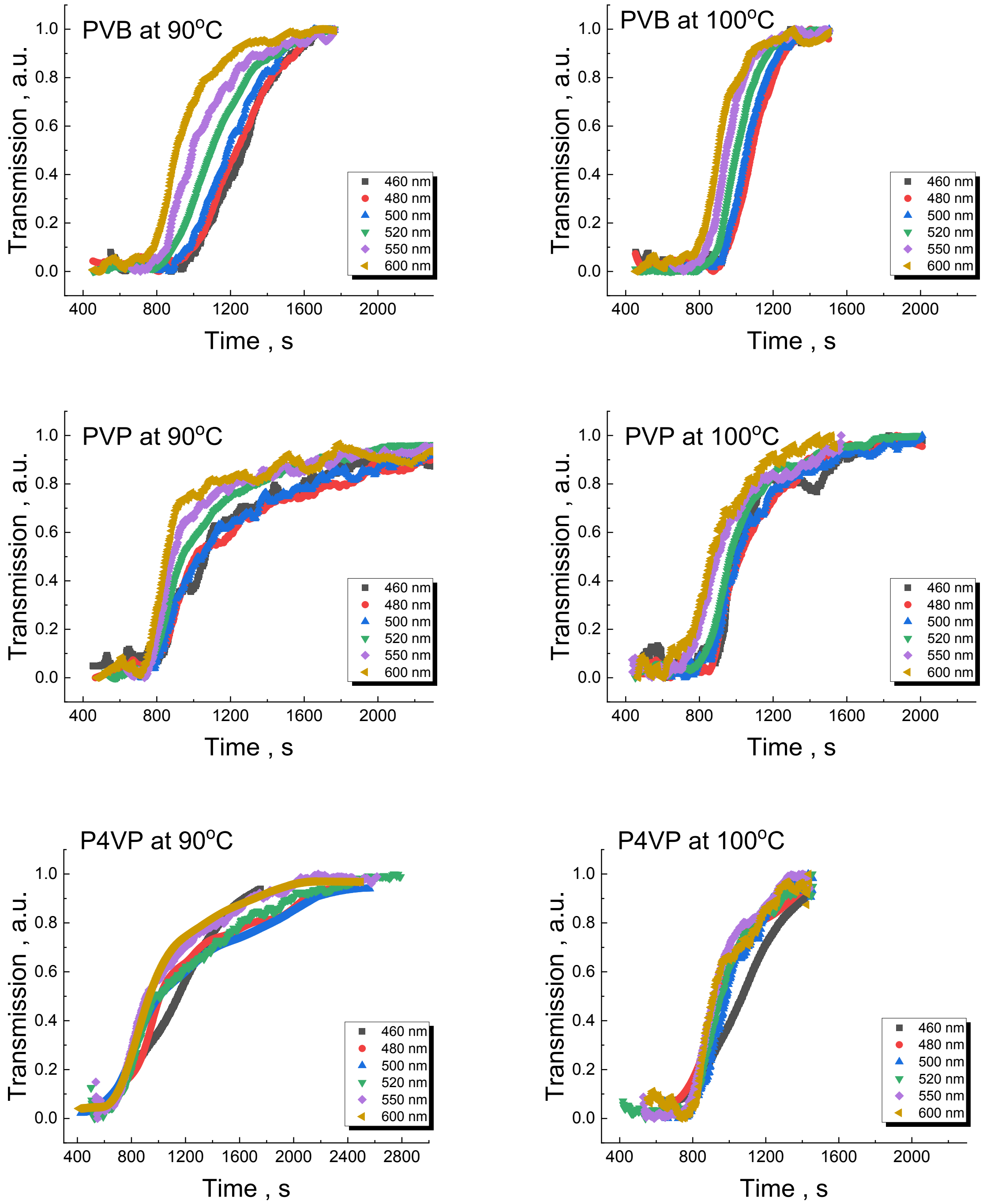
PVB at 90°C
PVB at 100°C
PVP at 90°C
PVP at 100°C
P4VP at 90°C
P4VP at 100°C
Transmission , a.u.
Time , s
460 nm
480 nm
500 nm
520 nm
550 nm
600 nm

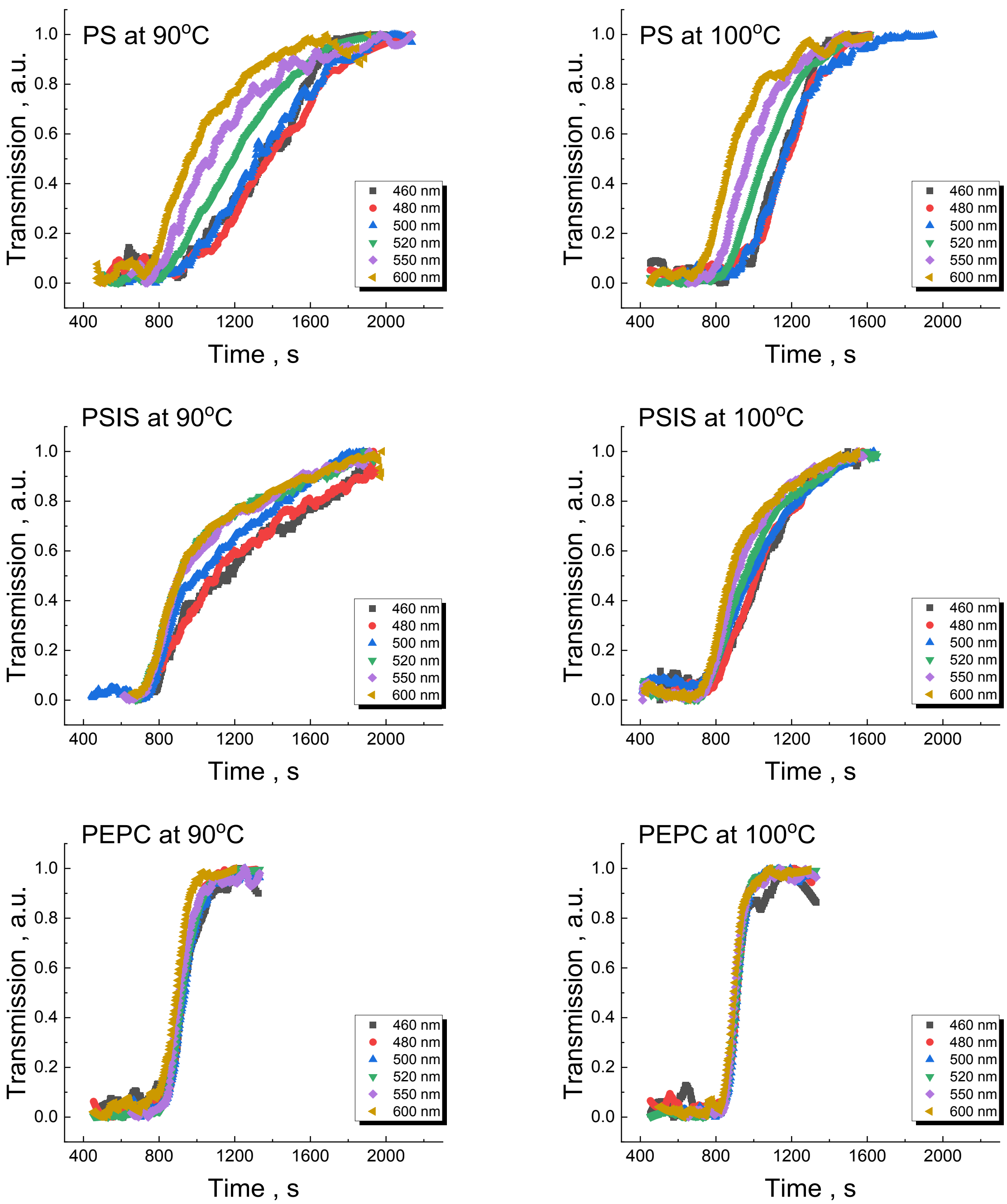

Figure S5. Comparison of normalized transmission recovery kinetics recorded on different wavelengths at 90 ºC (left panel) and 100 ºC (right panel) for studied polymers arranged according to their molecular weight (highest to smallest).

Dispersivity indexes (β) and time constants (τ) as obtained from the fitting to (5) and (6) are shown in Fig. S6 for *in situ* UV and thermal recovery absorbance kinetics recorded at different wavelengths.

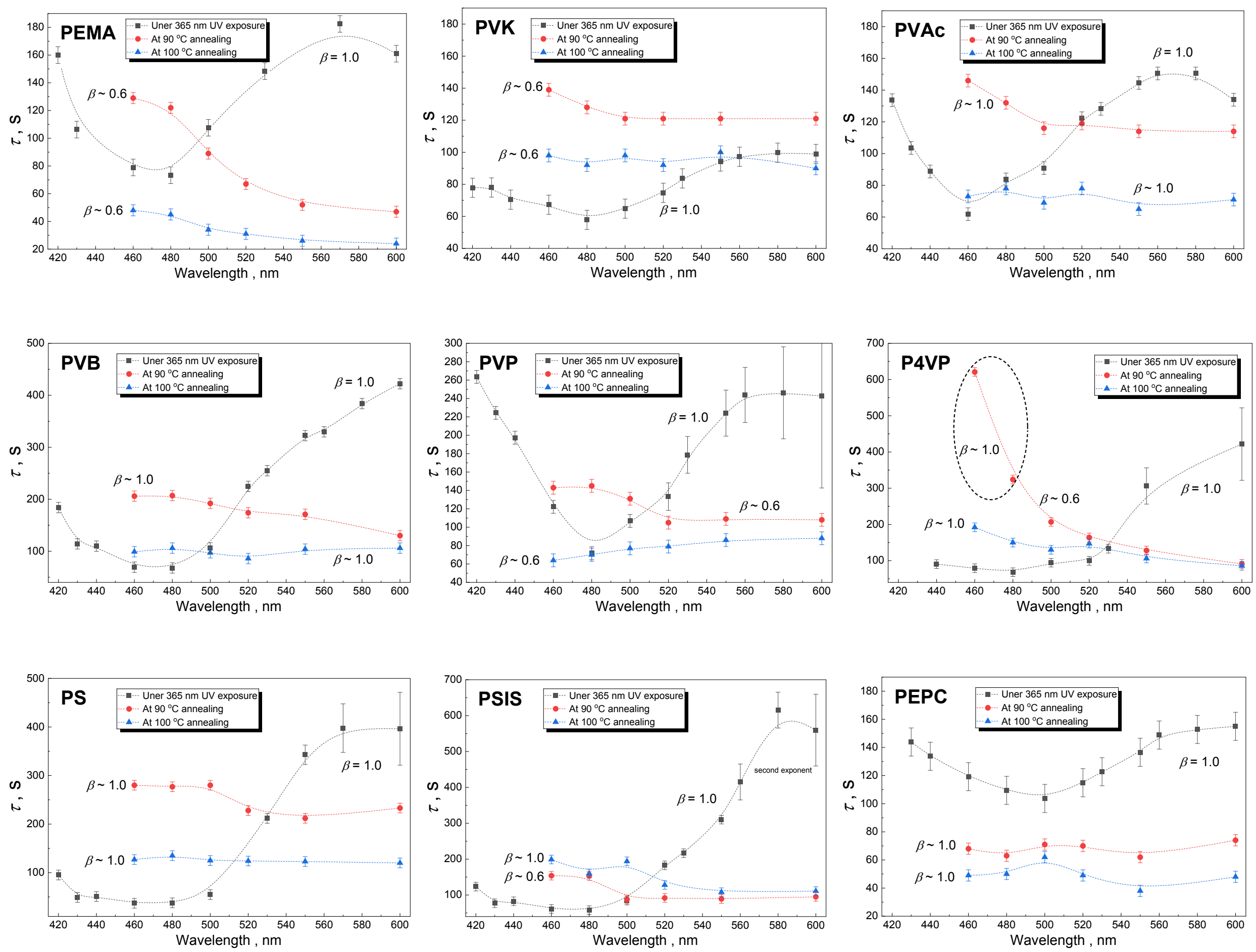

Figure S6. Comparison of time constants as obtained from fitting to (5) and (6) for *in situ* UV and thermal recovery absorbance kinetics, respectively, recorded at different wavelengths. The lines are drawn as a guide to the eyes.
